# Muscle–brain crosstalk mediated by exercise-induced myokines - insights from experimental studies

**DOI:** 10.3389/fphys.2024.1488375

**Published:** 2024-12-02

**Authors:** Magdalena Kostka, Julia Morys, Andrzej Małecki, Marta Nowacka-Chmielewska

**Affiliations:** Laboratory of Molecular Biology, Institute of Physiotherapy and Health Sciences, Academy of Physical Education, Katowice, Poland

**Keywords:** myokines, exercise, physical activity, muscle-brain crosstalk, rodent models

## Abstract

Over the past couple of decades, it has become apparent that skeletal muscles might be engaged in endocrine signaling, mostly as a result of exercise or physical activity in general. The importance of this phenomenon is currently studied in terms of the impact that exercise- or physical activity -induced signaling factors have, in the interaction of the “muscle-brain crosstalk.” So far, skeletal muscle-derived myokines were demonstrated to intercede in the connection between muscles and a plethora of various organs such as adipose tissue, liver, or pancreas. However, the exact mechanism of muscle-brain communication is yet to be determined. It is speculated that, in particular, brain-derived neurotrophic factor (BDNF), irisin, cathepsin B (CTSB), interleukin 6 (IL-6), and insulin-like growth factor-1 (IGF-1) partake in this crosstalk by promoting neuronal proliferation and synaptic plasticity, also resulting in improved cognition and ameliorated behavioral alterations. Researchers suggest that myokines might act directly on the brain parenchyma via crossing the blood-brain barrier (BBB). The following article reviews the information available regarding rodent studies on main myokines determined to cross the BBB, specifically addressing the association between exercise-induced myokine release and central nervous system (CNS) impairments. Although the hypothesis of skeletal muscles being critical sources of myokines seems promising, it should not be forgotten that the origin of these factors might vary, depending on the cell types engaged in their synthesis. Limited amount of research providing information on alterations in myokines expression in various organs at the same time, results in taking them only as circumstantial evidence on the way to determine the actual involvement of skeletal muscles in the overall state of homeostasis. The following article reviews the information available regarding rodent studies on main myokines determined to cross the BBB, specifically addressing the association between exercise-induced myokine release and CNS impairments.

## 1 Introduction

Understanding the potential of increased physical activity and exercise as vital components of a broadly understood healthy lifestyle, in disease prevention and treatment, is of increasing interest. Studies suggest that exercise-based interventions may have a beneficial effect on central nervous system (CNS) functionality. It was determined that exercising regularly might contribute to decreasing the risk of most CNS disorders, such as mood disorders and neurodegenerative diseases (Wang et al., 2024; Rai and Demontis, 2022). Improvement in mood and cognition (including memory and learning) in both patients and animal models of depression (Chen et al., 2017), Alzheimer’s disease (De la Rosa et al., 2020; Lourenco et al., 2019; Weinstein et al., 2014; Gaitán et al., 2021) or Parkinson’s disease (Zhou et al., 2017; Zhang et al., 2023) following exercise. The benefits of exercise have been determined to reshape the brain structure of depression patients, promote behavioral adaptation changes, and maintain the integrity of hippocampal regions (Zhao et al., 2020) or connectivity by enhancing neurogenesis and synaptic plasticity, and changing metabolism and vascular function (Cotman et al., 2007).

An improvement in behavior and cognitive abilities was determined to correspond with upregulated levels of neurotrophic factors and markers of synaptic plasticity, accompanied by downregulated proinflammatory mediators’ expression during exercise (Rai and Demontis, 2022; Mattson, 2012; Abdulghani et al., 2023). Many studies have examined the role of myokines in the context of beneficial effects of exercise on brain function, the aging process, and neurodegeneration. Myokines, muscle-secreted growth factors, and cytokines were suggested to play an important role in mediating the positive effects of exercise on the CNS (Rai and Demontis, 2022; Hutton et al., 2015; Farmer et al., 2004) via encompassing a variety of exercise-induced signaling molecules release, as a result of aerobic or strength training (Pedersen and Febbraio, 2008; Trillaud et al., 2023; Chow et al., 2022). Approximately a thousand proteins produced by skeletal muscles have been identified, but only parts have been well characterized (Pang et al., 2021; Florin et al., 2020). Therefore, skeletal muscles showed a significant endocrine capacity to impact many tissues and organs suggesting that also the CNS might be a target of muscle-initiated signaling. In addition to endocrine signaling through the circulation, direct muscle-to-nerve connections may also provide a route for signaling from skeletal muscle to the brain (Pedersen, 2019; Pedersen et al., 2007; Chen W. et al., 2021; Kim et al., 2019). Adipokines and hepatokines are also involved in the mediation of the beneficial effects caused by exercises, via promoting neurogenesis, synaptic plasticity, improving cognitive functions and energy metabolism, highlighting the existence of muscle-brain signaling (Trillaud et al., 2023; Lauretani et al., 2020; Vivar et al., 2013).

Acting by autocrine and paracrine signaling, myokines intercede in crosstalks between skeletal muscles and other organs, such as the brain (Hutton et al., 2015; Ding et al., 2006; Nakajima et al., 2010), adipose tissue (Boström et al., 2012; Tanimura et al., 2022), bones (Shahabi et al., 2021), liver (Nakajima et al., 2010; Tanimura et al., 2022; Chan et al., 2024), heart (Ho et al., 2018) or intestines (Lee et al., 2023; Liu et al., 2023). Their role is also to mediate metabolic processes, thereby contributing to the growth and regeneration of skeletal muscle cells (Rai and Demontis, 2022; Duzel et al., 2016; Jodeiri Farshbaf and Alviña, 2021), as well as enhancing muscle hypertrophy development (Erickson et al., 2013; Li et al., 2022; Delezie et al., 2019). Research shows that the level of myokines in muscles varies depending on their structure and specific function. In rodent studies, the greatest emphasis is put on the calf muscles (soleus, gastrocnemius) (Florin et al., 2020; Augusto et al., 2004), and the quadriceps muscles (Erickson et al., 2013; Li et al., 2022; Zakharova et al., 2021). It is also suggested that the type of incorporated training predisposes muscles to different myokine secretion profiles (Molanouri Shamsi et al., 2015). The endurance training was determined to cause an increase in IL-6 concentration in slow-twitch muscles (soleus) (Isanejad et al., 2015), while after strength training, IL-6 expression was comparable in both types of muscles: slow-twitch soleus and fast-twitch flexor hallucis longus (Molanouri Shamsi et al., 2015).

The exercise was determined to induce a favorable myokine profile and stimulate skeletal muscle cells to produce regulatory factors such as cytokines, hormones, growth factors, and exosomes (Chow et al., 2022; Chen W. et al., 2021; Blume and Royes, 2024; Pedersen, 2009; Vints et al., 2022). This beneficial myokine profile has been associated with effects on lipolysis and β-oxidation, glucose uptake and metabolism, or angiogenesis (Ji et al., 2024; So et al., 2014; Giudice and Taylor, 2017). Although some circulating factors cannot pass the blood-brain barrier (BBB), they still might influence the brain by binding to receptors located on the endothelial cells of the BBB (Zhang et al., 2018). As a result of increased physical activity, the following main myokines are secreted: interleukins (IL-4, -6, -7, -15) (Zakharova et al., 2021; Roca-Rivada et al., 2012), insulin-like growth factor-1 (IGF-1) (Erickson et al., 2013; Munive et al., 2016; Munive et al., 2019), brain-derived neurotrophic factor (BDNF) (Hutton et al., 2015; Ding et al., 2006; Farmer et al., 2004; Chan et al., 2024), fibroblast growth factor 21 (FGF21) (Mashili et al., 2011). Importantly, it has been established that irisin/FNDC5 (fibronectin-domain III containing 5), IGF-1, IL-6, cathepsin B (CTSB), and BDNF might cross the BBB, subsequently contributing to neuronal functioning improvement by modulating synaptic plasticity (Pedersen, 2019; Jodeiri Farshbaf and Alviña, 2021; Hashimoto et al., 2021; Ni et al., 2022; Jiang et al., 2020).

**FIGURE 1:**
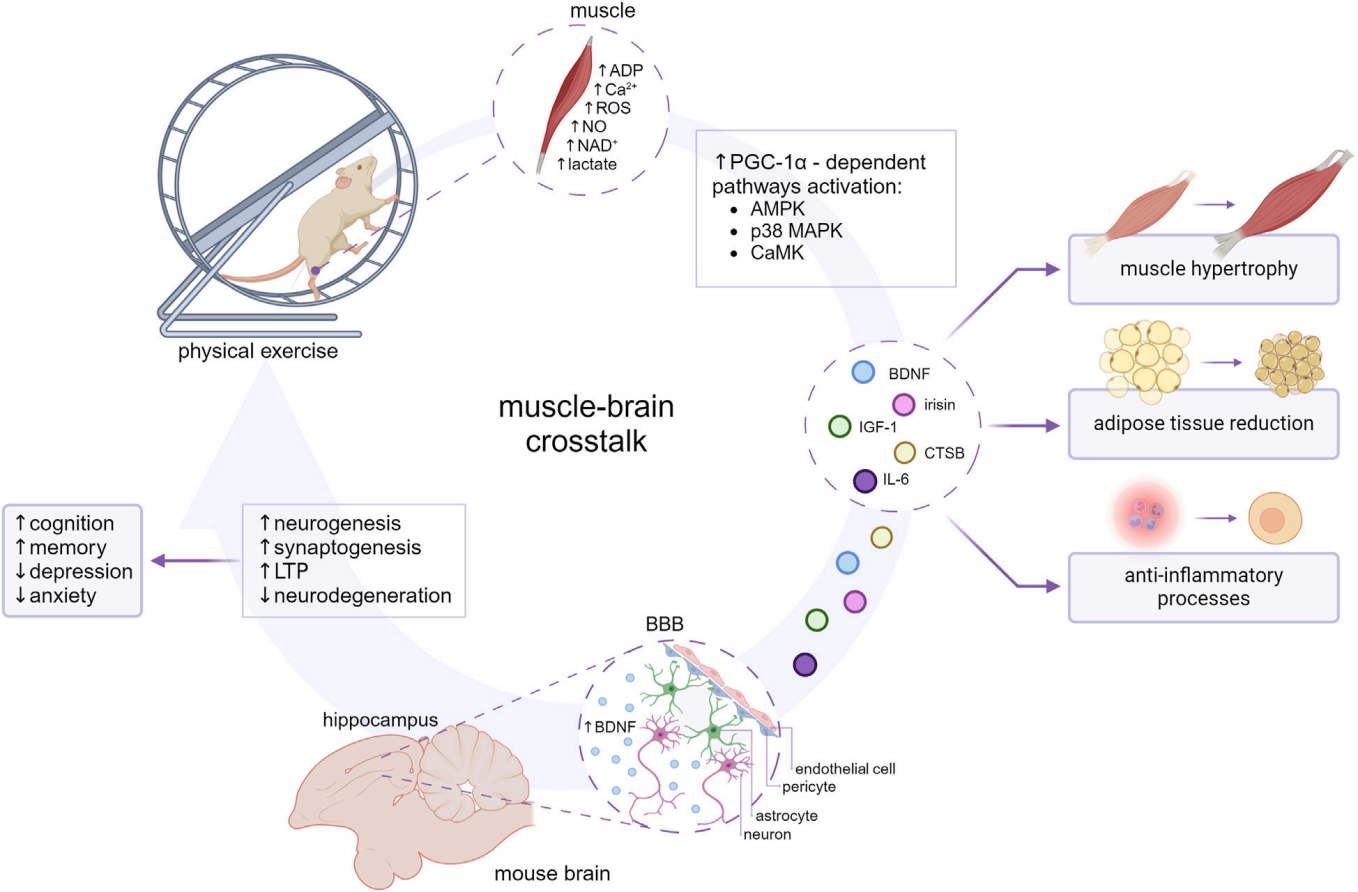
The impact of myokines on the muscle-brain crosstalk. The molecular mechanisms involved in the release of myokines seem to coincide with molecular adaptation to exercise and are largely mediated by transcription factors such as peroxisome proliferator-activated receptor (PPAR)-gamma coactivator-1α (PGC-1α). PGC-1α-dependent myokines (BDNF, irisin, CTSB, IL-6, IGF-1) are subsequently released into the blood circuitry and influence an array of metabolic processes. At the cellular level myokines might ameliorate inflammation, impact lipid and carbohydrate metabolism, and enhance angiogenesis and mitochondrial biogenesis. BDNF, CTSB, IL-6, IGF-1, and irisin are suggested to cross the BBB and reach the brain parenchyma, contributing to neurogenesis, synaptogenesis and long-term potentiation (LTP) enhancement, and consecutive improvement in cognition, memory, and behavioral alterations. Created with BioRender.com.

The following article reviews the information available regarding the main myokines determined to cross BBB, specifically addressing the association between exercise-induced myokine release and mood disorder development. This review summarizes only the results of experimental research that might support the role of those myokines in muscle-brain crosstalk (Figure 1).

## 2 Myokines as a messenger of skeletal muscle

Muscle contractions induce many physiological and metabolic adaptations in other organs, which are not mediated by the nervous system. Therefore, these processes might be mediated by myokines - factors expressed, produced, and secreted during exercise by muscle fibers (Pedersen, 2019; Pedersen et al., 2007; Chen W. et al., 2021; Kim et al., 2019). Although the myokines are generally characterized as cytokines released by myocytes, it should be emphasized that other muscle cell types were established with continuous cytokines’ expression as well (reviewed by Peake et al., 2015). *In vitro* studies on C2C12 myoblasts (Chan et al., 2024), L6 myoblasts (Moon et al., 2016), or myotubes provide insights into which myokines might be specific for which cell types. Based on the cell culture studies, myoblasts-mesodermal precursors of myocytes-were characterized with persistent expression of IL-8, IL-12, IL-15 or TGF- β. Moreover, the expression of myoblast-located myokines was found lower than in myotubes. Interestingly, some myokines were demonstrated to have fiber-specific expression. IL-6 expression was found the highest in type II muscle fibers as a result of endurance exercise. On the other hand, it was suggested that VEGF expression might be found mostly in the subsarcolemmal sarcoplasm of type I, IIa, and IIb muscle fibers after endurance exercise (reviewed by Peake et al. (2015)).

Experimental studies indicate that the communication between the brain and muscles is reshaped by exercise via the activity of many signaling pathways and the consequent modulation of the expression and secretion of several myokines with neuroprotective functions. Mutual interactions between myokines, especially BDNF and IGF-1, and the brain occur through pathways called the “muscle-brain axis” (Duzel et al., 2016; Lee et al., 2024; Burtscher et al., 2021) or the “muscle-brain crosstalk” (Pedersen, 2019; Chen W. et al., 2021; Lauretani et al., 2020; Blume and Royes, 2024; Ibeas et al., 2021). This connection is often observed when muscular damage (Li et al., 2019), and thus low levels of myokines, are associated with impaired cognitive functioning following sarcopenia (Blume and Royes, 2024; Jo et al., 2022; Choi et al., 2024; Markowska et al., 1998). Katz et al. (2022) established similarities in AKT (protein kinase B) and mTOR (mammalian target of rapamycin) pathways activity in the hypothalamus and muscles as a result of both, voluntary and forced exercise. An increase in AKT and a decrease in mTOR activity in muscles were also observed in the hypothalamus, supporting the idea of muscle-brain coordination existence. It was also confirmed by a study unveiling the impact of musclin on improved hypothalamic signaling and concomitant amelioration of depressive-like behaviors (Ataka et al., 2023). Moreover, irisin was found to act as a regulator of monoamine levels in subcortical brain regions, associated with vulnerability to developing neuropsychiatric disorders (Yardimci et al., 2023). Other evidence indicated a possible role of exercise-induced increased serum- and glucocorticoid-inducible kinase 1 (SGK1) expression and its neuroprotective features on improvement in cognition (Liu et al., 2024).

## 3 Exercise-induced myokine release regulation

The molecular mechanisms involved in the release of myokines seem to coincide with molecular adaptation to exercise and are largely mediated by transcription factors such as PGC-1α and PPAR-β, and metabolic sensors such as 5′-AMP activated kinase (AMPK). Recent studies showed that the activity of myokines is mostly regulated by signaling pathways related to the PGC-1α protein (Chan et al., 2024; Fan et al., 2017; Calvo et al., 2008). Its expression is induced by calcium ions released as a result of exercise, as well as by energy obtained from adipose tissue in cold environmental conditions/temperatures (Puigserver et al., 1998; Lin et al., 2002). The increase in PGC-1α concentration was observed as a result of strength training in both young (10 weeks) and middle-aged (50 weeks) rats (Jung et al., 2015). PGC-1α was suggested to induce mitochondrial biogenesis, mainly during aerobic training in slow-twitch muscles, while the PGC-1α4 release was established during strength training leading to muscle hypertrophy (Lin et al., 2002; Miller et al., 2019; Villena, 2015). An increase in the muscle PGC-1α alone, with no additional exercise interventions, did not induce such effects, as confirmed by studies on transgenic mice with overexpressed PGC-1α (Karlsson et al., 2019). Moreover, this overexpression was demonstrated to lead to metabolic disorders such as insulin resistance (Rai and Demontis, 2022; Karlsson et al., 2021). Mice overexpressing PGC-1α were characterized by increased profiles of cytochrome C, cytochrome oxidase II, and cytochrome IV oxidase (Mirebeau-Prunier et al., 2010; Wende et al., 2007), and showed improved performance functions such as increased peak oxygen uptake V_O2_ and covering longer distances at higher speeds while running on a treadmill (Calvo et al., 2008). Skeletal-muscle-specific knockout animals for PGC-1α were shown to suffer from a reduced endurance capacity as well as other signs of pathological inactivity (Handschin et al., 2007a; Handschin et al., 2007b). Increased expression of genes encoding oxidative phosphorylation enzymes was demonstrated in the gastrocnemius muscle, which indicated an improvement in lipid metabolism (Calvo et al., 2008; Wende et al., 2007). On the other hand, insulin sensitivity and glucose uptake were not changed in transgenic mice compared to control mice (Calvo et al., 2008).

In addition to PGC1α-mediated responses, a key component of exercise is the depletion of energy stores and the consequent activation of the AMPK, which senses AMP/ADP (adenosine monophosphate/adenosine diphosphate) concentrations. Mitochondria biogenesis, a highly coordinated process that requires multiple cellular events, including transcription of two genomes (nuclear and mitochondrial) (Hood et al., 1989; Cisterna et al., 2023), is strongly induced by the disturbance of metabolic homeostasis generated by exercise training. This dysregulation activates the main signaling pathways such as AMPK, MAPK (mitogen-activated protein kinase), PKA (protein kinase A), and also increased cytoplasmic free Ca^2+^, thereby impacting the expression of myokines (Hawley et al., 2014; Angulo et al., 2020; Reisman et al., 2024; Brooks, 2018). Changes in the mitochondrial oxidative capacity accompanied by an increase in the number of mitochondria and mitochondrial enzyme activity in response to exercise seem to be the main determinant (Laurens et al., 2020).

Here, we introduce some of the myokines that have been suggested to cross BBB and have frequently been linked to cognition, including BDNF, irisin, CTSB, IGF-1, and IL-6 (Table 1).

**TABLE 1:** List of animal studies that investigate exercise myokines expressed and/or secreted by skeletal muscle which act on muscle-brain tissue crosstalk.

Animal model			Exercise model		Effects of exercise on myokines			Reference
Animal	Sex	Age	Type	Duration	Serum	Muscle	Hippocampus	
PGC-1α overexpression mouse MCK-PGC-1α C57BL/6J	Both	12 and 44 weeks	Voluntary running	4 weeks	↑ PGC-1α	= PGC-1α	—	Karlsson et al. (2021)
Wild type rat Sprague-Dawley	Male	8 weeks	Voluntary running	4 weeks (every 2 days)	—	—	↑ BDNF	Hopkins and Bucci (2010)
BDNF KO mouse *bdnf* ^*flox/flox*^ C57BL/6J	Both	12– weeks	Treadmill running	1 day (single bout)	↓ BDNF	↓ *bdnf*	—	Delezie et al. (2019)
Wild type mouse NMRI	Male	Adult (no details data)	Treadmill running, LRT	8 weeks (3 days/week)	↑ irisin	↑ irisin	—	Momenzadeh et al. (2021)
Wild type mouse NMRI	Male	5 weeks	Treadmill running, LRT	8 weeks (3 days/week)	↑ irisin	↑ irisin	—	Shahabi et al. (2021)
Wild type mouse C57BL/6J	Male	5 weeks	Voluntary running	4 weeks	↑ CTSB	↑ CTSB *↑ ctsb*	*↑ ctsb*	Moon et al. (2016)
Wild type mouse C57BL/6J	Both	4 and 32 weeks	Treadmill running	16 weeks	—	↓ IL-6	—	Zakharova et al. (2021)
Wild type rat Wistar	Male	32 weeks	Treadmill running - downhill	8 weeks (5 days/week)	—	↑ IL-6	—	Isanejad et al. (2015)
Wild type rat Wistar	Male	adult (no details data)	LRT	1 day (single bout)	—	↑ IL-6	—	Molanouri Shamsi et al. (2015)
Wild type mouse C57BL/6J	Male	12 weeks	Treadmill running	2 weeks (7 days/week)	↑ IGF-1	—	= IGF-1	Llorens-Martín et al. (2010)
Wild type rat Sprague-Dawley	Male	7 weeks	Voluntary running	2 weeks	—	—	↑ IGF-1	Sølvsten et al. (2018)
Wild type mouse C57BL/6J	Male	48 weeks	Treadmill running, LRT	4 weeks (5 days/week)	—	↑ IGF-1	—	Li et al. (2022)
Wild type mouse C57BL/6J	Male	7 weeks	Voluntary running	5 weeks (7 days/week)	—	—	↑ IGF-1	Nakajima et al. (2010)
Wild type mouse C57BL/6J	Male	12 weeks	Treadmill running, LRT	4 weeks (5 days/week)	—	↑ IGF-1	—	Feng et al. (2022)
Wild type mouse C57BL/6J	Female	8 and 36 weeks	Treadmill running	2 weeks (5 days/week)	↑ IGF-1	—	↑ IGF-1	Munive et al. (2019)
Wild type mouse C57BL/6J	Both	8–9 weeks	Treadmill running	2 weeks	= IGF-1	—	↑ IGF-1	Munive et al. (2016)
Wild type rat Wistar	Male	10 and 50 weeks	LRT	8 weeks (every 3 days)	—	↑ IL-6 ↑ PGC-1	—	Jung et al. (2015)
BDNF KO mouse *bdnf* ^*flox/flox*^	Female	12 weeks	Treadmill running	4 weeks (5 days/week)	↓ BDNF ↓ PGC-1	—	—	Chan et al. (2024)
Wild type mouse C57BL/6J	Both	12 weeks	Swimming	5 weeks (5 days/week)	—	—	↑ irisin ↑ BDNF	Lourenco et al. (2019)
Wild type rat Sprague-Dawley	Male	Adult (no details data)	Swimming	6 days	—	—	↑ IGF-1 ↑ BDNF	Jiang et al. (2014)
Wild type mouse C57BL/6J	Male	6 weeks	Voluntary running	4 weeks	= BDNF = IGF-1	—	↑ *bdnf* ↑ IGF-1	Hutton et al. (2015)
Wild type rat Sprague-Dawley	Male	Adult (no details data)	Voluntary running	5 days	—	—	↑ IGF-1 ↑ BDNF ↑ pro-BDNF ↑ *igf-1* ↑ *bdnf*	Ding et al. (2006)

BDNF, brain-derived neurotrophic factor; CTSB, cathepsin B; IGF-1, insulin-like growth factor 1; IL-6, interleukin 6; LRT, ladder-based resistance training, PGC-1α - peroxisome-proliferator activated receptor γ coactivator 1α. Level of protein/gene expression: ↑ increase; ↓ decrease; = no change. KO, knockout; both - male and female.

## 4 Myokines linked with muscle-brain crosstalk

### 4.1 Brain-derived neurotrophic factor (BDNF)

BDNF is one of the most thoroughly studied neurotrophins and the most studied myokine. Its well-described functions as neurotrophin, include promoting the formation and maturation of neurons, enhancing neurogenesis and neuroregeneration, and reducing apoptosis through tropomyosin-related kinase receptor B (TrkB) (Gaitán et al., 2021; Rezaee et al., 2023; Yang et al., 2019). Its beneficial effect on cognitive functions is manifested by intensifying synaptic plasticity, leading to LTP (Vints et al., 2022; Zhou et al., 2023; Binder and Scharfman, 2004). The exercise-induced upregulation of hippocampal BDNF expression was confirmed in many animal studies (Duzel et al., 2016; Vints et al., 2022; Zhou et al., 2023; Sorrells et al., 2018; Leal et al., 2014; Lu et al., 2014; Jiang et al., 2014). The increased concentration of BDNF was noted in mice serum and hippocampus after exercise, while a greater increase was observed during breaks in exercise periods (Hutton et al., 2015; Ding et al., 2006; Farmer et al., 2004; Jo et al., 2022; Lei et al., 2024). Increased levels of BDNF in the hippocampus were also demonstrated in the offspring of mice running on a treadmill (Kim et al., 2024). Importantly, an exercise-induced increase in hippocampal BDNF expression was associated with functional changes, for instance, a reduction of anxiety- and depressive-like behaviors and improved cognition in voluntary-running rats (Hopkins and Bucci, 2010; Eldomiaty et al., 2017; Yau et al., 2012; Bechara and Kelly, 2013; Lee et al., 2012).

The neuronal mechanisms involved in exercise-induced release of neurotransmitters leading to the upregulation of *bdnf* expression in the brain are well known. During exercise, membrane depolarization and neurotransmitter binding lead to ligand-gated and voltage-gated Ca^2+^ channels (L-VGCC) activation at the cell membrane. Increased Ca^2+^ concentration activates signaling systems including the MAPK, the Ca^2+/^calmodulin-activated kinase (CAMK), and Ca^2+−^sensitive adenylate cyclase/PKA, leading to CREB (cAMP response element-binding protein) phosphorylation, and BDNF transcription. In addition to membrane depolarization, glutamatergic transmission mediated by AMPA (α-amino-3-hydroxy-5-methyl-4-isoxazolepropionic acid receptor) and NMDA receptors (*N*-methyl-D-aspartate receptor), plays an important role in this process (Nishijima et al., 2012).

Recently, it was proposed that cerebral mechanisms involved in exercise contribution to BDNF overexpression and concomitant positive effect on neurogenesis might be associated with exercise-induced metabolic factors (e.g., ketone bodies, lactate) and muscle-derived myokines (CTSB, irisin/FNDC5) (Moon et al., 2016).

CTSB and FNDC5 knockout mice showed decreased BDNF expression in the hippocampus and deteriorated cognitive functions, as demonstrated in a few behavioral tests: Morris Water Maze (MWM) (Moon et al., 2016), the Novel Object Recognition (NOR) (Lourenco et al., 2019), and the Open Field (Lourenco et al., 2019). Similarly, intraperitoneal administration of lactate with its inhibitor (MCT1/2) normalized the increase of BDNF in the hippocampus in mice subjected to exercise. Lower BDNF levels were associated with worsened memory in mice in the MWM test (Hayek et al., 2019) and NOR test (Hopkins and Bucci, 2010) compared to the control group.

Although BDNF is expressed primarily in the CNS, it might be also found in blood platelets (Radka et al., 1996; Karege et al., 2005), vascular endothelial cells (Bai et al., 2014), and organs associated with high metabolic activity, i.e., heart (Wang et al., 2018), kidneys (Tao et al., 2018), liver (Zhang et al., 2021), spleen (Prochnik et al., 2022), and skeletal muscles (Chan et al., 2024). As a result of exercise, the upregulation of BDNF in skeletal muscles was observed (Cuppini et al., 2007). Indeed, BDNF is differentially expressed in skeletal muscles depending on either homeostatic or pathological conditions (reviewed in Chevrel et al. (2006)). BDNF was linked with skeletal muscle regeneration. Its increased expression, followed by the activation and proliferation of satellite cells was observed after a muscle injury (Clow and Jasmin, 2010; Omura et al., 2005). Although many studies were associated with the role of BDNF in muscle development and function, the impact of muscle contraction on circulating BDNF levels is still understudied. One study suggests that based on an intravenous administration, BDNF might cross the BBB (Pan et al., 1998). Taken together, BDNF could be classified as a myokine, however, it is a matter of debate whether skeletal muscle could be a primary source of circulating BDNF levels. One of the proposed recent mechanisms involved the transport of BDNF in exosomes that could allow sustained release of BDNF in the brain (Yuan et al., 2017).

### 4.2 Irisin/FNDC5

Irisin/FNDC5, a glycosylated type 1 membrane protein, has been identified as an important exercise-regulated factor that induces major metabolic benefits (Boström et al., 2012). Circulating irisin affects systemic homeostasis by regulating osteogenesis (Estell et al., 2020), reducing adipose tissue (Momenzadeh et al., 2021), leading to muscle hypertrophy (Reza et al., 2017), and inducing neurogenesis (Wrann et al., 2013). During exercise, mainly strength training, irisin induces osteogenesis, by increasing the expression of osteopontin and inhibiting the production of sclerostin (Kim et al., 2018). Increased bone density (Shahabi et al., 2021) and higher concentrations of irisin in the blood circuitry were found in mice subjected to resistance training in comparison to aerobic training or external administration of this myokine (Shahabi et al., 2021; Momenzadeh et al., 2021). Interestingly, external administration of irisin did not affect the bone density (Shahabi et al., 2021). Primarily discovered as a secreted factor from skeletal muscle, irisin expression was shown to be dependent on the PGC-1α activity (Wrann et al., 2013). Wrann et al. found increased irisin secretion in the quadriceps and the hippocampus of voluntary running mice, suggesting that irisin might pass through the BBB (Wrann et al., 2013). In the hippocampus, irisin was determined to affect the Akt, Erk, cAMP/PKA/CREB pathways, inducing an increase in BDNF expression with concomitant PGC-1α upregulation, thus influencing neuronal proliferation (Rai and Demontis, 2022; Momenzadeh et al., 2021; Wrann et al., 2013). Following the exercise, Val66Met polymorphism was associated with reduced expression of brain FNDC5 and BDNF in mice (Ieraci et al., 2016). Extending those findings is a study by Lourenco et al. showing the reduced brain levels of irisin in AD (Alzheimer’s disease) mouse models and further, stating that irisin might be a novel mediator of the beneficial effects of exercise on synapse function and memory in AD models (Lourenco et al., 2019). In addition, recent data shows that elevation of the circulating irisin by its peripheral delivery improved cognitive function in transgenic AD mouse models (APP/PS1), and even in wild-type mice (Islam et al., 2021). PFF α-syn mice (non-transgenic model of Parkinson’s disease (PD)) injected with irisin showed improved motor functions, reduced tyrosine hydroxylase activity, loss of dopamine receptors and dopaminergic neurons, and reduced amount of residing α-synuclein (Kam et al., 2022).

### 4.3 Cathepsin B (CTSB)

Lysosomal cysteine protease CTSB is ubiquitously expressed throughout the body (Turk et al., 2012). Depending on the pH of the environment, it assumes the function of exopeptidase (acidic pH) or endopeptidase (neutral pH) (Schmitz et al., 2019; Yoon et al., 2022). CTSB normally functions in lysosomes thereby degrading proteins and maintaining cellular homeostasis. However, in pathological conditions, it is translocated to the cytosol, where it activates inflammatory processes, and induces cell death (Wang et al., 2023; Lambeth et al., 2021). CTSB is highly expressed in tumors thus it is used as a biomarker for imaging (Chen X. et al., 2021). CTSB located in lysosomes was determined to promote autophagy and immune response by trafficking TNF-α (tumor necrosis factor α)-containing vesicles (Ni et al., 2022; Ha et al., 2008). Autophagy is currently being studied in psychiatric conditions. It is well established that cathepsins are engaged in disease progression (Moon et al., 2016; Matthews et al., 2023). Kindy et al. demonstrated that CTSB knockout mice in the model of AD exhibit memory impairments (Kindy et al., 2012) and that the inhibition of CTSB promotes the degradation of β-amyloid in AD (Choi et al., 2013).

CTSB, recently discovered as myokine, was reported to increase in plasma in response to aerobic exercising in animal models (including voluntary wheel running mice and treadmill training monkeys) (Moon et al., 2016). Passing through the BBB, CTSB promotes BDNF expression in the hippocampus, contributing to neurogenesis and improving memory (Rai and Demontis, 2022; Jiang et al., 2020; Ibeas et al., 2021). It also initiates the growth of neurites and axons, thereby contributing to the nervous system development (Jiang et al., 2020). In a study by Moon et al. (2016) involving voluntarily running male mice, CTSB expression in the hippocampus was upregulated compared to the sedentary group. The authors suggested CTSB as a mediator of the effects of exercise on cognition. Consistently, CTSB concentration in the plasma and gastrocnemius muscles was also increased. Furthermore, intravenous administration of CTSB to CTSB knockout mice enhanced BDNF and doublecortin (DCX) expression in adult hippocampal progenitor cells (Moon et al., 2016).

Interestingly, CTSB activity was found to be tissue-dependent. Gornicka et al. suggested that overexpression of CTSB in adipose tissue might lead to increased lysosomal membrane permeabilization, promote inflammation, and upregulate autophagy. This initial event leads to reactive oxygen species (ROS) overproduction, mitochondrial dysfunction, and at the end to adipocyte death and macrophage infiltration (Gornicka et al., 2012). Dysregulation at the cellular level is associated with chronic inflammation in adipose tissue leading to glucose intolerance, hepatic steatosis, insulin resistance, cardiovascular disease, and type 2 diabetes (Araujo et al., 2018). Research by Daneshyar et al. (2023) determined increased CTSB expression in adipose tissue, in mice fed with HFD. However, these findings were not observed after subjecting mice to exercise. These inconsistent results might demonstrate the tissue-specific CTSB activity supported by studies covering autophagy-upregulated CTSB expression is concomitant with increased autophagy in adipose tissue. As a result of exercise, HFD-induced CTSB increase was lower than in the non-exercised group. The following outcomes might be associated with Bcl-2 (B-cell lymphoma 2 protein) being a substrate for CTSB synthesis, the expression of which is downregulated by exercise (reviewed in Araujo et al., 2018). Taken together, these outcomes may indicate the role of exercise in CTSB-mediated obesity prevention.

### 4.4 Interleukin-6 (IL-6)

IL-6 is a pleiotropic cytokine with a broad spectrum of biological activity (Rose-John, 2012), produced by various cells, including immune cells, renal mesangial cells, adipocytes, muscle cells, and cancer cells (Kontny and Maśliński et al., 2009). It is involved in the immune response and inflammation during several disease progression (Sharif et al., 2018). In classic signaling, IL-6 binds to complex receptor IL-6Rα with glycoprotein gp130 expression on the cell membrane surface, majoring in immune cells (macrophages, lymphocytes) or skeletal myocytes (Sharif et al., 2018; Rose-John et al., 2017; Lin et al., 2023). By engaging soluble IL-6R (sIL-6R) and membrane-bound gp130, IL-6 induces a pro-inflammatory effect by trans-signaling (Rose-John et al., 2017; Petersen and Pedersen, 2006).

Regarding IL-6 impact on the CNS, it was determined to contribute to promoting neuronal survival (März et al., 1998; Pervaiz and Hoffman-Goetz, 2012). IL-6 and its receptors were found in brain endothelial cells (Eskilsson et al., 2014) and in brain structures like the hippocampus, cortex, thalamus, and clastrum (Vallières et al., 1997; Gadient and Otten, 1994; Schöbitz et al., 1993; Baier et al., 2009). It was suggested to cross the BBB (Banks et al., 1994) and to promote neurogenesis (influencing both neurons and glial cells) (Vallières et al., 2002). On the other hand, IL-6 might act completely opposing actions triggering either neuronal survival after injury or causing neuronal degeneration and cell death in disorders, such as AD (Kummer et al., 2021).

As a result of prolonged exercise, increased production and release of IL-6 in skeletal muscles classified this cytokine as myokine. Additionally, muscles are its important targets (reviewed in Muñoz-Cánoves et al., 2013). Souza et al. (2017) demonstrated the protective effects of exercising on the BBB permeability in an experimental model of autoimmune neuroinflammation. Experimental autoimmune encephalomyelitis (EAE) mouse model subjected to exercise showed decreased BBB permeability and downregulated cytokines expression, such as IFN-γ, IL-6, IL-17, and IL-1b (Souza et al., 2017). IL-6 levels were increased in treadmill-running mice while TNF-α levels were decreased in the hippocampus (Pervaiz and Hoffman-Goetz, 2012).

Muscle-induced IL-6 is responsible for metabolic effects and tissue regeneration (Kelly et al., 2009; Ahsan et al., 2022; Pedersen et al., 2007; Rose-John et al., 2017). Kelly et al. suggested that IL-6 released by gastrocnemius muscle in swimming rats might initiate the AMPK pathway and promote lipolysis, and glycogenolysis (Kelly et al., 2004). Jung et al. determined exercising induced an increase in IL-6 expression in the tibialis muscle in rats. The increase in slow-twitch muscle-derived IL-6 was accompanied by a decrease in TNF-α after endurance training in rodents (Isanejad et al., 2015; Jung et al., 2015). Similar observations were made in a study by Zuo et al., covering inflammation induced in muscles, after subjecting rats to running on a treadmill (Zuo et al., 2019). Importantly, it was proposed that exercise-induced IL-6 may be responsible for the long-term anti-inflammatory effects via enhancing epithelial cell division and growth and limiting apoptosis (Rose-John, 2012; Phillips and Fahimi, 2018; Kistner et al., 2022; Petersen and Pedersen, 2006).

It should be emphasized that IL-6 is highly synthesized and released post-exercise while insulin release is enhanced (Pedersen and Febbraio, 2008). However, IL-6 is also associated with obesity and insulin resistance (Pedersen and Febbraio, 2008). An increased higher IL-6 mRNA expression in the skeletal muscle was found in high-fat diet rats than in the control rats, and importantly it could be reduced to a much lower level with aerobic exercise (Gopalan et al., 2021). IL-6 was also shown to increase glucose uptake through mediating translocation of GLUT4 (glucose transporter 4) receptors in many tissues including muscle (Carey et al., 2006; Gopalan et al., 2021) and brain (Hussein et al., 2021; Vannucci et al., 1998). Moreover, Pearson-Leary et al. demonstrated that increasing hippocampal GLUT4 expression contributed to improving memory in exercising rats (Pearson-Leary and McNay, 2016). In addition, IL-6 was found to reduce TNF-α levels and enhance insulin release acting via the AMPK pathway (Oh et al., 2016). Thus, the level of IL-6 is closely related to the glucose metabolism that occurs in muscles during exercise (Isanejad et al., 2015; Helge et al., 2003; Pedersen, 2009).

### 4.5 Insulin-like growth factor –1 (IGF-1)

IGF-1, a polypeptide similar structurally and functionally to insulin, acts as a mediator of most tissue effects of growth hormone (Vajdos et al., 2001; Laron, 2001). It is associated with neuronal development, neurogenesis, and synaptogenesis (Cheng et al., 2003; Llorens-Martín et al., 2010; Mir et al., 2017; Ransome and Hannan, 2013; Glasper et al., 2010), and shows neuroprotective features following nerve injury (Kim et al., 2021) or stroke (Zhang et al., 2013). The IGF-1 expression in the brain was decreased throughout the lifespan in the aging rats (Frutos et al., 2007), contributing to a greater susceptibility to oxidative stress (Holzenberger et al., 2003) and increased microglial activity inducing inflammation (Kohman et al., 2012).

Although muscle-derived IGF-1 is not detected in circulation, it is considered as another representative myokine. An increase in serum IGF-1 levels was observed during break periods in exercising (Ransome and Hannan, 2013). It was established that exercise-induced IGF-1 release occurs mainly after strength and resistance training (Li et al., 2022; Feng et al., 2022). IGF-1 was established to partake in anabolic processes, activate myogenesis (Feng et al., 2022), stimulate protein synthesis, and promote muscle hypertrophy (Feng et al., 2022; Yoshida and Delafontaine, 2020). The increase in muscle density might be generated via the IGF-1R/PI3K/Akt signaling pathway (Li et al., 2022; Feng et al., 2022; Kraemer et al., 2017), the stimulation of which was proposed to alleviate skeletal muscle atrophy (Yoshida and Delafontaine, 2020). PGC-1α was found to be an important element in combating insulin resistance by affecting growth hormone and IGF-1 (Kang and Li Ji, 2012). Growth hormone-induced IGF-1 release in the pituitary gland was shown to induce angiogenesis (Norling et al., 2020) and modulate the formation of pro-BDNF, leading to an increase in BDNF in the hippocampus (Ding et al., 2006). Blocking the IGF-1 receptor significantly reversed the exercise-induced increase in the BDNF expression, suggesting that the effects of IGF-1 may be caused by interceding the conversion of pro-BDNF to BDNF (Ding et al., 2006). Furthermore, exercise-induced alterations in the expression of IGF-1 in the brain were associated with synaptic and cognitive plasticity (Ding et al., 2006), and improvement in neurogenesis and memory (Trillaud et al., 2023; Ding et al., 2006). Increased IGF-1 expression in the hippocampus was observed in voluntarily running rats (Sølvsten et al., 2018). However, other studies determined a decrease in IGF-1 in male mice subjected to forced exercise, unlike females, who showed an increase in protein expression in the hippocampus (Munive et al., 2016). Munive et al. suggested that estradiol (E2) might stimulate IGF-1 uptake. Ovariectomized, exercising female mice exhibited reduced levels of IGF-1 in the hippocampus compared to the sedentary females.

Exercise-induced improvement of memory and learning, especially in animals previously subjected to chronic stress, was associated with increased expression of IGF-1 (Ding et al., 2006), FNDC5, and BDNF (Choi et al., 2024; Jiang et al., 2014) in the hippocampus (Nakajima et al., 2010; Dief et al., 2015; Phillips and Fahimi, 2018). In the MWM test, Llorens-Martin et al. demonstrated memory impairment in mice with blocked IGF-1 receptors (Llorens-Martín et al., 2010). On the other hand, treadmill-running mice with IGF-1 receptor inhibition showed exercise-induced cell proliferation compared to the sedentary group (Glasper et al., 2010).

## 5 Conclusion and future research directions

Exercise has been suggested as a potent and robust non-pharmacological intervention for improving cognitive function, including learning and memory. Factors produced and released by skeletal muscles, including myokines, growth factors, hormones, and cytokines are considered in mediating the beneficial influence of exercise on CNS functioning, appetite, and metabolism, thus supporting the existence of a muscle-brain crosstalk. Animal studies indicate that communication between the brain and skeletal muscles is reshaped by prolonged exercise via myokine-related signaling pathways and the consequent modulation of the expression and secretion of several myokines with neuroprotective functions. Notably, the myokine profile was determined dependent on the variety of myokine-secreting cells and the exercise regimen. Whether direct or indirect, BDNF, CTSB, IL-6, and IGF-1 have been shown to facilitate the cross-talk between the brain and muscles, but the relative contribution of each myokine to the beneficial effects of exercise on the CNS remains complex.

Considering that the concept of myokines is a relatively new direction in studies covering the molecular background of exercise, the main direction of future research conceptualizations should be to identify and describe further molecules that might be classified as this type of exerkines. Pending the analysis of the muscle-brain axis in terms of bidirectional interaction via exerkine release being its direct effect, it should not be forgotten that myokines are not molecules of muscle origin only, although the hypothesis of skeletal muscles being critical sources of myokines seems promising. A limited amount of research provides information on alterations in myokines expression in both components of the axis at once. Therefore, if possible, the future directions of experimental studies on the muscle-brain axis should involve the comparison of the myokine expression in both engaged tissues, completed with the serum levels of studied exerkines. Moreover, the interaction between the brain and muscles has been examined in diverse animal models regarding experimental studies. If unified, could be a promising measure of stating more precise conclusions in terms of molecular mechanisms underlying the crosstalk and addressing the broad population. The division of specific types of training applied should be emphasized as well. Recent studies determined that the outcomes of myokines release are dependent on the type of either aerobic or resistance training that animals were exposed to. Since the muscle-brain interaction is not solely existent, interactions based on other types of exerkines are also involved in the crosstalk functionality. Particular attention should be paid to the effect of myokines on adipose tissue. Given the beneficial effects of exercise on adipose tissue remodeling, research efforts should be placed on the identification of novel exercise-induced myokines that may control adipose tissue metabolism and function. Not only do myokines partake in exercise-induced increased metabolism but also, the communication between myokines and adipokines have been found significant in improving cognition.

The terms of exercise and contingent myokines release should be drawn to researchers’ attention, given the facility of incorporating exercise as an alternative approach to decreasing the risk of CNS-linked disorders encourages further exploration.

## Glossary

AD: Alzheimer’s disease
ADP: adenosine diphosphate
AKT: protein kinase B
AMP: adenosine monophosphate
AMPA: α-amino-3-hydroxy-5-methyl-4-isoxazolepropionic acid receptor
AMPK: 5′-AMP-activated kinase adenosine monophosphate (AMP)-dependent kinase
BBB: blood-brain barrier
Bcl-2: B-cell lymphoma 2 protein
BDNF: brain-derived neurotrophic factor
CaMK: calcium/calmodulin-dependent protein kinase
CNS: central nervous system
CREB: cAMP response element-binding protein
CTSB: cathepsin B
DCX: doublecortin
E2: estradiol
FGF21: fibroblast growth factor 21
FNDC5: fibronectin-domain III containing 5
GLUT4: glucose transporter 4
HFD: high fat diet
IGF-1: insulin-like growth factor 1
IL-6: interleukin 6
L-VGCC: ligand-gated and voltage-gated Ca^2+^ channels
LTP: long-term potentiation
mTOR: mammalian target of rapamycin
MWM: Morris water maze
NMDA: N-methyl-D-aspartate receptor
NOR: novel object recognition
p38 MAPK: p38 mitogen-activated protein kinase
PD: Parkinson’s disease
PGC-1α: peroxisome-proliferator activated receptor γ coactivator 1α
PKA: protein kinase A
PPAR γ: peroxisome-proliferator activated receptor γ
ROS: reactive oxygen species
SGK1: serum- and glucocorticoid-inducible kinase 1
TNF-α: tumor necrosis factor α
TrkB: tropomyosin-related kinase receptor B.

## References

[B1] Abdulghani A. Poghosyan M. Mehren A. Philipsen A. Anderzhanova E. (2023). Neuroplasticity to autophagy cross-talk in a therapeutic effect of physical exercises and irisin in ADHD. Front. Mol. Neurosci. 15, 997054. 10.3389/fnmol.2022.997054 36776770 PMC9909442

[B2] Ahsan M. Garneau L. Aguer C. (2022). The bidirectional relationship between AMPK pathway activation and myokine secretion in skeletal muscle: how it affects energy metabolism. Front. physiology 13, 1040809. 10.3389/fphys.2022.1040809 PMC9721351 36479347

[B3] Angulo J. El Assar M. Álvarez-Bustos A. Rodríguez-Mañas L. (2020). Physical activity and exercise: strategies to manage frailty. Redox Biol. 35, 101513. 10.1016/j.redox.2020.101513 32234291 PMC7284931

[B4] Araujo T. F. Cordeiro A. V. Vasconcelos D. A. A. Vitzel K. F. Silva V. R. R. (2018). The role of cathepsin B in autophagy during obesity: a systematic review. Life Sci. 209, 274–281. 10.1016/j.lfs.2018.08.024 30107168

[B5] Ataka K. Asakawa A. Iwai H. Kato I. (2023). Musclin prevents depression-like behavior in male mice by activating urocortin 2 signaling in the hypothalamus. Front. Endocrinol. 14, 1288282. 10.3389/fendo.2023.1288282 PMC10728487 38116320

[B6] Augusto V. Padovani C. R. Eduardo G. Campos R. (2004). Skeletal muscle fiber types in C57BL6J mice. J. Morphol. Sci. 21 (2), 89–94.

[B7] Bai X. Yilin C. Qi X. Cai D. (2014). Single-cell analysis for BDNF and TrkB receptors in cardiac microvascular endothelial cells. Bio-medical Mater. Eng. 24 (6), 2257–2264. 10.3233/BME-141038 25226925

[B8] Baier P. C. May U. Scheller J. Rose-John S. Schiffelholz T. (2009). Impaired hippocampus-dependent and -independent learning in IL-6 deficient mice. Behav. brain Res. 200 (1), 192–196. 10.1016/j.bbr.2009.01.013 19378383

[B9] Banks W. A. Kastin A. J. Gutierrez E. G. (1994). Penetration of interleukin-6 across the murine blood-brain barrier. Neurosci. Lett. 179 (1-2), 53–56. 10.1016/0304-3940(94)90933-4 7845624

[B10] Bechara R. G. Kelly Á. M. (2013). Exercise improves object recognition memory and induces BDNF expression and cell proliferation in cognitively enriched rats. Behav. brain Res. 245, 96–100. 10.1016/j.bbr.2013.02.018 23439217

[B11] Binder D. K. Scharfman H. E. (2004). Brain-derived neurotrophic factor. Growth factors Chur, Switz. 22 (3), 123–131. 10.1080/08977190410001723308 PMC2504526 15518235

[B12] Blume G. R. Royes L. F. F. (2024). Peripheral to brain and hippocampus crosstalk induced by exercise mediates cognitive and structural hippocampal adaptations. Life Sci. 352, 122799. 10.1016/j.lfs.2024.122799 38852798

[B13] Boström P. Wu J. Jedrychowski M. P. Korde A. Ye L. Lo J. C. (2012). A PGC1-α-dependent myokine that drives brown-fat-like development of white fat and thermogenesis. Nature 481 (7382), 463–468. 10.1038/nature10777 22237023 PMC3522098

[B14] Brooks G. A. (2018). The science and translation of lactate shuttle theory. Cell metab. 27 (4), 757–785. 10.1016/j.cmet.2018.03.008 29617642

[B15] Burtscher J. Millet G. P. Place N. Kayser B. Zanou N. (2021). The muscle-brain Axis and neurodegenerative diseases: the key role of mitochondria in exercise-induced neuroprotection. Int. J. Mol. Sci. 22 (12), 6479. 10.3390/ijms22126479 34204228 PMC8235687

[B16] Calvo J. A. Daniels T. G. Wang X. Paul A. Lin J. Spiegelman B. M. (2008). Muscle-specific expression of PPARgamma coactivator-1alpha improves exercise performance and increases peak oxygen uptake. J. Appl. physiology (Bethesda, Md. 1985) 104 (5), 1304–1312. 10.1152/japplphysiol.01231.2007 18239076

[B17] Carey A. L. Steinberg G. R. Macaulay S. L. Thomas W. G. Holmes A. G. Ramm G. (2006). Interleukin-6 increases insulin-stimulated glucose disposal in humans and glucose uptake and fatty acid oxidation *in vitro* via AMP-activated protein kinase. Diabetes 55 (10), 2688–2697. 10.2337/db05-1404 17003332

[B18] Chan W. S. Ng C. F. Pang B. P. S. Hang M. Tse M. C. L. Iu E. C. Y. (2024). Exercise-induced BDNF promotes PPARδ-dependent reprogramming of lipid metabolism in skeletal muscle during exercise recovery. Sci. Signal. 17 (828), eadh2783. 10.1126/scisignal.adh2783 38502732 PMC11022078

[B19] Chen L. Zhou C. Tan C. Wang F. Gao Y. Huang C. (2017). Stereological study on the positive effect of running exercise on the capillaries in the Hippocampus in a depression model. Front. Neuroanat. 11, 93. 10.3389/fnana.2017.00093 29204111 PMC5698265

[B20] Chen W. Wang L. You W. Shan T. (2021). Myokines mediate the cross talk between skeletal muscle and other organs. J. Cell. physiology 236 (4), 2393–2412. 10.1002/jcp.30033 32885426

[B21] Chen X. Ren X. Zhu Y. Fan Z. Zhang L. Liu Z. (2021). Cathepsin B-activated fluorescent and photoacoustic imaging of tumor. Anal. Chem. 93 (27), 9304–9308. 10.1021/acs.analchem.1c02145 34181407

[B22] Cheng C. M. Mervis R. F. Niu S. L. Salem N. Jr Witters L. A. Tseng V. (2003). Insulin-like growth factor 1 is essential for normal dendritic growth. J. Neurosci. Res. 73 (1), 1–9. 10.1002/jnr.10634 12815703

[B23] Chevrel G. Hohlfeld R. Sendtner M. (2006). The role of neurotrophins in muscle under physiological and pathological conditions. Muscle and nerve 33 (4), 462–476. 10.1002/mus.20444 16228973

[B24] Cho K. Yoon S. Y. Choi J. E. Kang H. J. Jang H. Y. Kim D. H. (2013). CA-074Me, a cathepsin B inhibitor, decreases APP accumulation and protects primary rat cortical neurons treated with okadaic acid. Neurosci. Lett. 548, 222–227. 10.1016/j.neulet.2013.05.056 23748042

[B25] Choi J. W. Jo S. W. Kim D. E. Paik I. Y. Balakrishnan R. (2024). Aerobic exercise attenuates LPS-induced cognitive dysfunction by reducing oxidative stress, glial activation, and neuroinflammation. Redox Biol. 71, 103101. 10.1016/j.redox.2024.103101 38408409 PMC10904279

[B26] Chow L. S. Gerszten R. E. Taylor J. M. Pedersen B. K. van Praag H. Trappe S. (2022). Exerkines in health, resilience and disease. Nat. Rev. Endocrinol. 18 (5), 273–289. 10.1038/s41574-022-00641-2 35304603 PMC9554896

[B27] Cisterna B. Lofaro F. D. Lacavalla M. A. Boschi F. Malatesta M. Quaglino D. (2023). Aged gastrocnemius muscle of mice positively responds to a late onset adapted physical training. Front. cell Dev. Biol. 11, 1273309. 10.3389/fcell.2023.1273309 38020923 PMC10679468

[B28] Clow C. Jasmin B. J. (2010). Brain-derived neurotrophic factor regulates satellite cell differentiation and skeltal muscle regeneration. Mol. Biol. cell 21 (13), 2182–2190. 10.1091/mbc.e10-02-0154 20427568 PMC2893983

[B29] Cotman C. W. Berchtold N. C. Christie L. A. (2007). Exercise builds brain health: key roles of growth factor cascades and inflammation. Trends Neurosci. 30 (9), 464–472. 10.1016/j.tins.2007.06.011 17765329

[B30] Cuppini R. Sartini S. Agostini D. Guescini M. Ambrogini P. Betti M. (2007). Bdnf expression in rat skeletal muscle after acute or repeated exercise. Arch. Ital. Biol. 145 (2), 99–110. 17639782

[B31] Daneshyar S. Tavoosidana G. Bahmani M. Basir S. S. Delfan M. Laher I. (2023). Combined effects of high fat diet and exercise on autophagy in white adipose tissue of mice. Life Sci. 314, 121335. 10.1016/j.lfs.2022.121335 36587790

[B32] De la Rosa A. Olaso-Gonzalez G. Arc-Chagnaud C. Millan F. Salvador-Pascual A. García-Lucerga C. (2020). Physical exercise in the prevention and treatment of Alzheimer's disease. J. sport health Sci. 9 (5), 394–404. 10.1016/j.jshs.2020.01.004 32780691 PMC7498620

[B33] Delezie J. Weihrauch M. Maier G. Tejero R. Ham D. J. Gill J. F. (2019). BDNF is a mediator of glycolytic fiber-type specification in mouse skeletal muscle. Proc. Natl. Acad. Sci. U. S. A. 116 (32), 16111–16120. 10.1073/pnas.1900544116 31320589 PMC6690026

[B34] Dief A. E. M Samy D. I Dowedar F. (2015). Impact of exercise and vitamin B1 intake on hippocampal brain-derived neurotrophic factor and spatial memory performance in a rat model of stress. J. Nutr. Sci. vitaminology 61 (1), 1–7. 10.3177/jnsv.61.1 25994133

[B35] Ding Q. Vaynman S. Akhavan M. Ying Z. Gomez-Pinilla F. (2006). Insulin-like growth factor I interfaces with brain-derived neurotrophic factor-mediated synaptic plasticity to modulate aspects of exercise-induced cognitive function. Neuroscience 140 (3), 823–833. 10.1016/j.neuroscience.2006.02.084 16650607

[B36] Duzel E. van Praag H. Sendtner M. (2016). Can physical exercise in old age improve memory and hippocampal function? Brain a J. neurology 139 (Pt 3), 662–673. 10.1093/brain/awv407 PMC4766381 26912638

[B37] Eldomiaty M. A. Almasry S. M. Desouky M. K. Algaidi S. A. (2017). Voluntary running improves depressive behaviours and the structure of the hippocampus in rats: a possible impact of myokines. Brain Res. 1657, 29–42. 10.1016/j.brainres.2016.12.001 27919728

[B38] Erickson K. I. Gildengers A. G. Butters M. A. (2013). Physical activity and brain plasticity in late adulthood. Dialogues Clin. Neurosci. 15 (1), 99–108. 10.31887/DCNS.2013.15.1/kerickson 23576893 PMC3622473

[B39] Eskilsson A. Mirrasekhian E. Dufour S. Schwaninger M. Engblom D. Blomqvist A. (2014). Immune-induced fever is mediated by IL-6 receptors on brain endothelial cells coupled to STAT3-dependent induction of brain endothelial prostaglandin synthesis. J. Neurosci. official J. Soc. Neurosci. 34 (48), 15957–15961. 10.1523/JNEUROSCI.3520-14.2014 PMC6608482 25429137

[B40] Estell E. G. Le P. T. Vegting Y. Kim H. Wrann C. Bouxsein M. L. (2020). Irisin directly stimulates osteoclastogenesis and bone resorption *in vitro* and *in vivo* . eLife 9, e58172. 10.7554/eLife.58172 32780016 PMC7444909

[B41] Fan W. Waizenegger W. Lin C. S. Sorrentino V. He M. X. Wall C. E. (2017). PPARδ promotes running endurance by preserving glucose. Cell metab. 25 (5), 1186–1193.e4. 10.1016/j.cmet.2017.04.006 28467934 PMC5492977

[B42] Farmer J. Zhao X. van Praag H. Wodtke K. Gage F. H. Christie B. R. (2004). Effects of voluntary exercise on synaptic plasticity and gene expression in the dentate gyrus of adult male Sprague-Dawley rats *in vivo* . Neuroscience 124 (1), 71–79. 10.1016/j.neuroscience.2003.09.029 14960340

[B43] Feng L. Li B. Xi Y. Cai M. Tian Z. (2022). Aerobic exercise and resistance exercise alleviate skeletal muscle atrophy through IGF-1/IGF-1R-PI3K/Akt pathway in mice with myocardial infarction. Am. J. physiology. Cell physiology 322 (2), C164–C176. 10.1152/ajpcell.00344.2021 34852207

[B44] Florin A. Lambert C. Sanchez C. Zappia J. Durieux N. Tieppo A. M. (2020). The secretome of skeletal muscle cells: a systematic review. Osteoarthr. Cartil. open 2 (1), 100019. 10.1016/j.ocarto.2019.100019 36474563 PMC9718214

[B45] Frutos M. G. Cacicedo L. Méndez C. F. Vicent D. González M. Sánchez-Franco F. (2007). Pituitary alterations involved in the decline of growth hormone gene expression in the pituitary of aging rats. journals gerontology. Ser. A, Biol. Sci. Med. Sci. 62 (6), 585–597. 10.1093/gerona/62.6.585 17595414

[B46] Gadient R. A. Otten U. (1994). Expression of interleukin-6 (IL-6) and interleukin-6 receptor (IL-6R) mRNAs in rat brain during postnatal development. Brain Res. 637 (1-2), 10–14. 10.1016/0006-8993(94)91211-4 8180786

[B47] Gaitán J. M. Moon H. Y. Stremlau M. Dubal D. B. Cook D. B. Okonkwo O. C. (2021). Effects of aerobic exercise training on systemic biomarkers and cognition in late middle-aged adults at risk for alzheimer's disease. Front. Endocrinol. 12, 660181. 10.3389/fendo.2021.660181 PMC8173166 34093436

[B48] Giudice J. Taylor J. M. (2017). Muscle as a paracrine and endocrine organ. Curr. Opin. Pharmacol. 34, 49–55. 10.1016/j.coph.2017.05.005 28605657 PMC5808999

[B49] Glasper E. R. Llorens-Martin M. V. Leuner B. Gould E. Trejo J. L. (2010). Blockade of insulin-like growth factor-I has complex effects on structural plasticity in the hippocampus. Hippocampus 20 (6), 706–712. 10.1002/hipo.20672 19603528

[B50] Gopalan V. Yaligar J. Michael N. Kaur K. Anantharaj R. Verma S. K. (2021). A 12-week aerobic exercise intervention results in improved metabolic function and lower adipose tissue and ectopic fat in high-fat diet fed rats. Biosci. Rep. 41 (1), BSR20201707. 10.1042/BSR20201707 33432988 PMC7846962

[B51] Gornicka A. Fettig J. Eguchi A. Berk M. P. Thapaliya S. Dixon L. J. (2012). Adipocyte hypertrophy is associated with lysosomal permeability both *in vivo* and *in vitro*: role in adipose tissue inflammation. Am. J. physiology. Endocrinol. metabolism 303 (5), E597–E606. 10.1152/ajpendo.00022.2012 PMC3468510 22739104

[B52] Ha S. D. Martins A. Khazaie K. Han J. Chan B. M. Kim S. O. (2008). Cathepsin B is involved in the trafficking of TNF-alpha-containing vesicles to the plasma membrane in macrophages. J. Immunol. Baltim. Md. 1950 181 (1), 690–697. 10.4049/jimmunol.181.1.690 18566436

[B53] Handschin C. Chin S. Li P. Liu F. Maratos-Flier E. Lebrasseur N. K. (2007b). Skeletal muscle fiber-type switching, exercise intolerance, and myopathy in PGC-1alpha muscle-specific knock-out animals. J. Biol. Chem. 282 (41), 30014–30021. 10.1074/jbc.M704817200 17702743

[B54] Handschin C. Choi C. S. Chin S. Kim S. Kawamori D. Kurpad A. J. (2007a). Abnormal glucose homeostasis in skeletal muscle-specific PGC-1alpha knockout mice reveals skeletal muscle-pancreatic beta cell crosstalk. J. Clin. investigation 117 (11), 3463–3474. 10.1172/JCI31785 PMC2000810 17932564

[B55] Hashimoto T. Tsukamoto H. Ando S. Ogoh S. (2021). Effect of exercise on brain health: the potential role of lactate as a myokine. Metabolites 11 (12), 813. 10.3390/metabo11120813 34940571 PMC8709217

[B56] Hawley J. A. Hargreaves M. Joyner M. J. Zierath J. R. (2014). Integrative biology of exercise. Cell 159 (4), 738–749. 10.1016/j.cell.2014.10.029 25417152

[B57] Hayek L. E. Khalifeh M. Zibara V. Abi Assaad R. Emmanuel N. Karnib N. (2019). Lactate mediates the effects of exercise on learning and memory through SIRT1-dependent activation of hippocampal brain-derived neurotrophic factor (BDNF). J. Neurosci. official J. Soc. Neurosci. 39 (13), 2369–2382. 10.1523/JNEUROSCI.1661-18.2019 PMC6435829 30692222

[B58] Helge J. W. Stallknecht B. Pedersen B. K. Galbo H. Kiens B. Richter E. A. (2003). The effect of graded exercise on IL-6 release and glucose uptake in human skeletal muscle. J. physiology 546 (Pt 1), 299–305. 10.1113/jphysiol.2002.030437 PMC2342463 12509497

[B59] Ho M. Y. Wen M. S. Yeh J. K. Hsieh I. C. Chen C. C. Hsieh M. J. (2018). Excessive irisin increases oxidative stress and apoptosis in murine heart. Biochem. biophysical Res. Commun. 503 (4), 2493–2498. 10.1016/j.bbrc.2018.07.005 30208516

[B60] Holzenberger M. Dupont J. Ducos B. Leneuve P. Géloën A. Even P. C. (2003). IGF-1 receptor regulates lifespan and resistance to oxidative stress in mice. Nature 421 (6919), 182–187. 10.1038/nature01298 12483226

[B61] Hood D. A. Zak R. Pette D. (1989). Chronic stimulation of rat skeletal muscle induces coordinate increases in mitochondrial and nuclear mRNAs of cytochrome-c-oxidase subunits. Eur. J. Biochem. 179 (2), 275–280. 10.1111/j.1432-1033.1989.tb14551.x 2537205

[B62] Hopkins M. E. Bucci D. J. (2010). BDNF expression in perirhinal cortex is associated with exercise-induced improvement in object recognition memory. Neurobiol. Learn. Mem. 94 (2), 278–284. 10.1016/j.nlm.2010.06.006 20601027 PMC2930914

[B63] Hussein A. M. Awadalla A. Abbas K. M. Sakr H. F. Elghaba R. Othman G. (2021). Chronic valproic acid administration enhances oxidative stress, upregulates IL6 and downregulates Nrf2, Glut1 and Glut4 in rat's liver and brain. Neuroreport 32 (10), 840–850. 10.1097/WNR.0000000000001663 34050116

[B64] Hutton C. P. Déry N. Rosa E. Lemon J. A. Rollo C. D. Boreham D. R. (2015). Synergistic effects of diet and exercise on hippocampal function in chronically stressed mice. Neuroscience 308, 180–193. 10.1016/j.neuroscience.2015.09.005 26358368

[B65] Ibeas K. Herrero L. Mera P. Serra D. (2021). Hypothalamus-skeletal muscle crosstalk during exercise and its role in metabolism modulation. Biochem. Pharmacol. 190, 114640. 10.1016/j.bcp.2021.114640 34087244

[B66] Ieraci A. Madaio A. I. Mallei A. Lee F. S. Popoli M. (2016). Brain-derived neurotrophic factor Val66Met human polymorphism impairs the beneficial exercise-induced neurobiological changes in mice. Neuropsychopharmacol. official Publ. Am. Coll. Neuropsychopharmacol. 41 (13), 3070–3079. 10.1038/npp.2016.120 PMC5101555 27388329

[B67] Isanejad A. Saraf Z. H. Mahdavi M. Gharakhanlou R. Shamsi M. M. Paulsen G. (2015). The effect of endurance training and downhill running on the expression of IL-1β, IL-6, and TNF-α and HSP72 in rat skeletal muscle. Cytokine 73 (2), 302–308. 10.1016/j.cyto.2015.03.013 25863030

[B68] Islam M. R. Valaris S. Young M. F. Haley E. B. Luo R. Bond S. F. (2021). Exercise hormone irisin is a critical regulator of cognitive function. Nat. Metab. 3 (8), 1058–1070. 10.1038/s42255-021-00438-z 34417591 PMC10317538

[B69] Ji M. Cho C. Lee S. (2024). Acute effect of exercise intensity on circulating FGF-21, FSTL-1, cathepsin B, and BDNF in young men. J. Exerc. Sci. Fit. 22 (1), 51–58. 10.1016/j.jesf.2023.11.002 38074189 PMC10698539

[B70] Jiang M. Meng J. Zeng F. Qing H. Hook G. Hook V. (2020). Cathepsin B inhibition blocks neurite outgrowth in cultured neurons by regulating lysosomal trafficking and remodeling. J. Neurochem. 155 (3), 300–312. 10.1111/jnc.15032 32330298 PMC7581626

[B71] Jiang P. Dang R. L. Li H. D. Zhang L. H. Zhu W. Y. Xue Y. (2014). The impacts of swimming exercise on hippocampal expression of neurotrophic factors in rats exposed to chronic unpredictable mild stress. Evidence-based complementary Altern. Med. eCAM 2014, 729827. 10.1155/2014/729827 PMC4244932 25477997

[B72] Jo D. Yoon G. Kim O. Y. Song J. (2022). A new paradigm in sarcopenia: cognitive impairment caused by imbalanced myokine secretion and vascular dysfunction. Biomed. and Pharmacother. = Biomedecine and Pharmacother. 147, 112636. 10.1016/j.biopha.2022.112636 35051857

[B73] Jodeiri Farshbaf M. Alviña K. (2021). Multiple roles in neuroprotection for the exercise derived myokine irisin. Front. aging Neurosci. 13, 649929. 10.3389/fnagi.2021.649929 33935687 PMC8086837

[B74] Jung S. Ahn N. Kim S. Byun J. Joo Y. Kim S. (2015). The effect of ladder-climbing exercise on atrophy/hypertrophy-related myokine expression in middle-aged male Wistar rats. J. physiological Sci. JPS 65 (6), 515–521. 10.1007/s12576-015-0388-1 PMC10717129 26223833

[B75] Kam T. I. Park H. Chou S. C. Van Vranken J. G. Mittenbühler M. J. Kim H. (2022). Amelioration of pathologic α-synuclein-induced Parkinson's disease by irisin. Proc. Natl. Acad. Sci. U. S. A. 119 (36), e2204835119. 10.1073/pnas.2204835119 36044549 PMC9457183

[B76] Kang C. Li Ji L. (2012). Role of PGC-1α signaling in skeletal muscle health and disease. Ann. N. Y. Acad. Sci. 1271 (1), 110–117. 10.1111/j.1749-6632.2012.06738.x 23050972 PMC3499658

[B77] Karege F. Bondolfi G. Gervasoni N. Schwald M. Aubry J. M. Bertschy G. (2005). Low brain-derived neurotrophic factor (BDNF) levels in serum of depressed patients probably results from lowered platelet BDNF release unrelated to platelet reactivity. Biol. psychiatry 57 (9), 1068–1072. 10.1016/j.biopsych.2005.01.008 15860348

[B78] Karlsson L. González-Alvarado M. N. Motalleb R. Blomgren K. Börjesson M. Kuhn H. G. (2019). Constitutive PGC-1α overexpression in skeletal muscle does not protect from age-dependent decline in neurogenesis. Sci. Rep. 9 (1), 12320. 10.1038/s41598-019-48795-w 31444397 PMC6707251

[B79] Karlsson L. González-Alvarado M. N. Motalleb R. Wang Y. Wang Y. Börjesson M. (2021). Constitutive PGC-1α overexpression in skeletal muscle does not contribute to exercise-induced neurogenesis. Mol. Neurobiol. 58 (4), 1465–1481. 10.1007/s12035-020-02189-6 33200398 PMC7932943

[B80] Katz A. Gonen M. Shahar Y. Roichman A. Lerrer B. Cohen H. Y. (2022). Hypothalamus-muscle parallel induction of metabolic pathways following physical exercise. Front. Neurosci. 16, 897005. 10.3389/fnins.2022.897005 35928013 PMC9344923

[B81] Kelly M. Gauthier M. S. Saha A. K. Ruderman N. B. (2009). Activation of AMP-activated protein kinase by interleukin-6 in rat skeletal muscle: association with changes in cAMP, energy state, and endogenous fuel mobilization. Diabetes 58 (9), 1953–1960. 10.2337/db08-1293 19502419 PMC2731526

[B82] Kelly M. Keller C. Avilucea P. R. Keller P. Luo Z. Xiang X. (2004). AMPK activity is diminished in tissues of IL-6 knockout mice: the effect of exercise. Biochem. biophysical Res. Commun. 320 (2), 449–454. 10.1016/j.bbrc.2004.05.188 15219849

[B83] Kim D. J. Cho S. Y. Kim S. U. Jo D. W. Hwang H. I. Shin H. K. (2021). IGF-1 protects neurons in the cortex and subventricular zone in a periventricular leucomalacia model. vivo (Athens, Greece) 35 (1), 307–312. 10.21873/invivo.12260 PMC7880788 33402478

[B84] Kim H. Wrann C. D. Jedrychowski M. Vidoni S. Kitase Y. Nagano K. (2018). Irisin mediates effects on bone and fat via αV integrin receptors. Cell 175 (7), 1756–1768.e17. 10.1016/j.cell.2018.10.025 30550785 PMC6298040

[B85] Kim S. Choi J. Y. Moon S. Park D. H. Kwak H. B. Kang J. H. (2019). Roles of myokines in exercise-induced improvement of neuropsychiatric function. Pflugers Archiv Eur. J. physiology 471 (3), 491–505. 10.1007/s00424-019-02253-8 30627775

[B86] Kim T. W. Park S. S. Kim S. H. Kim M. K. Shin M. S. Kim S. H. (2024). Exercise before pregnancy exerts protective effect on prenatal stress-induced impairment of memory, neurogenesis, and mitochondrial function in offspring. J. Exerc. rehabilitation 20 (1), 2–10. 10.12965/jer.2448068.034 PMC10902695 38433854

[B87] Kindy M. S. Yu J. Zhu H. El-Amouri S. S. Hook V. Hook G. R. (2012). Deletion of the cathepsin B gene improves memory deficits in a transgenic ALZHeimer's disease mouse model expressing AβPP containing the wild-type β-secretase site sequence. J. Alzheimer's Dis. JAD 29 (4), 827–840. 10.3233/JAD-2012-111604 22337825 PMC4309289

[B88] Kistner T. M. Pedersen B. K. Lieberman D. E. (2022). Interleukin 6 as an energy allocator in muscle tissue. Nat. Metab. 4 (2), 170–179. 10.1038/s42255-022-00538-4 35210610

[B89] Kohman R. A. DeYoung E. K. Bhattacharya T. K. Peterson L. N. Rhodes J. S. (2012). Wheel running attenuates microglia proliferation and increases expression of a proneurogenic phenotype in the hippocampus of aged mice. Brain, Behav. Immun. 26 (5), 803–810. 10.1016/j.bbi.2011.10.006 22056294 PMC3275652

[B90] Kontny E. Maśliński W. (2009). Interleukina 6 – znaczenie biologiczne i rola w patogenezie reumatoidalnego zapalenia stawów. Reumatologia 47 (1), 24–33.

[B91] Kraemer W. J. Ratamess N. A. Nindl B. C. (2017). Recovery responses of testosterone, growth hormone, and IGF-1 after resistance exercise. J. Appl. physiology (Bethesda, Md, 1985) 122 (3), 549–558. 10.1152/japplphysiol.00599.2016 27856715

[B92] Kummer K. K. Zeidler M. Kalpachidou T. Kress M. (2021). Role of IL-6 in the regulation of neuronal development, survival and function. Cytokine 144, 155582. 10.1016/j.cyto.2021.155582 34058569

[B93] Lambeth T. R. Dai Z. Zhang Y. Julian R. R. (2021). A two-trick pony: lysosomal protease cathepsin B possesses surprising ligase activity. RSC Chem. Biol. 2 (2), 606–611. 10.1039/d0cb00224k 34291207 PMC8291735

[B94] Laron Z. (2001). Insulin-like growth factor 1 (IGF-1): a growth hormone. Mol. Pathol. MP 54 (5), 311–316. 10.1136/mp.54.5.311 11577173 PMC1187088

[B95] Laurens C. Bergouignan A. Moro C. (2020). Exercise-released myokines in the control of energy metabolism. Front. physiology 11, 91. 10.3389/fphys.2020.00091 PMC7031345 32116795

[B96] Lauretani F. Longobucco Y. Ferrari Pellegrini F. De Iorio A. M. Fazio C. Federici R. (2020). Comprehensive model for physical and cognitive frailty: current organization and unmet needs. Front. Psychol. 11, 569629. 10.3389/fpsyg.2020.569629 33324282 PMC7725681

[B97] Leal G. Comprido D. Duarte C. B. (2014). BDNF-induced local protein synthesis and synaptic plasticity. Neuropharmacology 76 (Pt C), 639–656. 10.1016/j.neuropharm.2013.04.005 23602987

[B98] Lee H. Kim S. Y. Lim Y. (2024). Solanum melongena extract supplementation protected skeletal muscle and brain damage by regulation of BDNF/PGC1α/irisin pathway via brain function-related myokines in high-fat diet induced obese mice. J. Nutr. Biochem. 124, 109537. 10.1016/j.jnutbio.2023.109537 38030047

[B99] Lee J. Y. Shin S. K. Bae H. R. Ji Y. Park H. J. Kwon E. Y. (2023). The animal protein hydrolysate attenuates sarcopenia via the muscle-gut axis in aged mice. Biomed. and Pharmacother. = Biomedecine and Pharmacother. 167, 115604. 10.1016/j.biopha.2023.115604 37804811

[B100] Lee M. C. Okamoto M. Liu Y. F. Inoue K. Matsui T. Nogami H. (2012). Voluntary resistance running with short distance enhances spatial memory related to hippocampal BDNF signaling. J. Appl. physiology (Bethesda, Md, 1985) 113 (8), 1260–1266. 10.1152/japplphysiol.00869.2012 22936723

[B101] Lei Z. Mozaffaritabar S. Kawamura T. Koike A. Kolonics A. Kéringer J. (2024). The effects of long-term lactate and high-intensity interval training (HIIT) on brain neuroplasticity of aged mice. Heliyon 10 (2), e24421. 10.1016/j.heliyon.2024.e24421 38293399 PMC10826720

[B102] Li B. Feng L. Wu X. Cai M. Yu J. J. Tian Z. (2022). Effects of different modes of exercise on skeletal muscle mass and function and IGF-1 signaling during early aging in mice. J. Exp. Biol. 225 (21), jeb244650. 10.1242/jeb.244650 36205111

[B103] Li G. Zhang L. Wang D. Aiqudsy L. Jiang J. X. Xu H. (2019). Muscle-bone crosstalk and potential therapies for sarco-osteoporosis. J. Cell. Biochem. 120 (9), 14262–14273. 10.1002/jcb.28946 31106446 PMC7331460

[B104] Lin J. Wu H. Tarr P. T. Zhang C. Y. Wu Z. Boss O. (2002). Transcriptional co-activator PGC-1 alpha drives the formation of slow-twitch muscle fibres. Nature 418 (6899), 797–801. 10.1038/nature00904 12181572

[B105] Lin W. Song H. Shen J. Wang J. Yang Y. Yang Y. (2023). Functional role of skeletal muscle-derived interleukin-6 and its effects on lipid metabolism. Front. physiology 14, 1110926. 10.3389/fphys.2023.1110926 PMC10405179 37555019

[B106] Liu X. Hu Q. Xu T. Yuan Q. Hu Q. Hu N. (2023). Fndc5/irisin deficiency leads to dysbiosis of gut microbiota contributing to the depressive-like behaviors in mice. Brain Res. 1819, 148537. 10.1016/j.brainres.2023.148537 37591459

[B107] Liu Y. Ye Q. Dai Y. Hu J. Chen J. Dong J. (2024). Integrating analysis of mRNA expression profiles indicates Sgk1 as a key mediator in muscle-brain crosstalk during resistance exercise. Biochem. biophysical Res. Commun. 719, 150075. 10.1016/j.bbrc.2024.150075 38749087

[B108] Llorens-Martín M. Torres-Alemán I. Trejo J. L. (2010). Exercise modulates insulin-like growth factor 1-dependent and -independent effects on adult hippocampal neurogenesis and behaviour. Mol. Cell. Neurosci. 44 (2), 109–117. 10.1016/j.mcn.2010.02.006 20206269

[B109] Lourenco M. V. Frozza R. L. de Freitas G. B. Zhang H. Kincheski G. C. Ribeiro F. C. (2019). Exercise-linked FNDC5/irisin rescues synaptic plasticity and memory defects in Alzheimer's models. Nat. Med. 25 (1), 165–175. 10.1038/s41591-018-0275-4 30617325 PMC6327967

[B110] Lu B. Nagappan G. Lu Y. (2014). BDNF and synaptic plasticity, cognitive function, and dysfunction. Handb. Exp. Pharmacol. 220, 223–250. 10.1007/978-3-642-45106-5_9 24668475

[B111] Markowska A. L. Mooney M. Sonntag W. E. (1998). Insulin-like growth factor-1 ameliorates age-related behavioral deficits. Neuroscience 87 (3), 559–569. 10.1016/s0306-4522(98)00143-2 9758223

[B112] März P. Cheng J. G. Gadient R. A. Patterson P. H. Stoyan T. Otten U. (1998). Sympathetic neurons can produce and respond to interleukin 6. Proc. Natl. Acad. Sci. U. S. A. 95 (6), 3251–3256. 10.1073/pnas.95.6.3251 9501249 PMC19728

[B113] Mashili F. L. Austin R. L. Deshmukh A. S. Fritz T. Caidahl K. Bergdahl K. (2011). Direct effects of FGF21 on glucose uptake in human skeletal muscle: implications for type 2 diabetes and obesity. Diabetes/metabolism Res. Rev. 27 (3), 286–297. 10.1002/dmrr.1177 21309058

[B114] Matthews I. Birnbaum A. Gromova A. Huang A. W. Liu K. Liu E. A. (2023). Skeletal muscle TFEB signaling promotes central nervous system function and reduces neuroinflammation during aging and neurodegenerative disease. Cell Rep. 42 (11), 113436. 10.1016/j.celrep.2023.113436 37952157 PMC10841857

[B115] Mattson M. P. (2012). Energy intake and exercise as determinants of brain health and vulnerability to injury and disease. Cell metab. 16 (6), 706–722. 10.1016/j.cmet.2012.08.012 23168220 PMC3518570

[B116] Miller K. N. Clark J. P. Anderson R. M. (2019). Mitochondrial regulator PGC-1a-Modulating the modulator. Curr. Opin. Endocr. metabolic Res. 5, 37–44. 10.1016/j.coemr.2019.02.002 PMC6690794 31406949

[B117] Mir S. Cai W. Carlson S. W. Saatman K. E. Andres D. A. (2017). IGF-1 mediated neurogenesis involves a novel RIT1/akt/sox2 cascade. Sci. Rep. 7 (1), 3283. 10.1038/s41598-017-03641-9 28607354 PMC5468318

[B118] Mirebeau-Prunier D. Le Pennec S. Jacques C. Gueguen N. Poirier J. Malthiery Y. (2010). Estrogen-related receptor alpha and PGC-1-related coactivator constitute a novel complex mediating the biogenesis of functional mitochondria. FEBS J. 277 (3), 713–725. 10.1111/j.1742-4658.2009.07516.x 20067526

[B119] Molanouri Shamsi M. Hassan Z. M. Quinn L. S. Gharakhanlou R. Baghersad L. Mahdavi M. (2015). Time course of IL-15 expression after acute resistance exercise in trained rats: effect of diabetes and skeletal muscle phenotype. Endocrine 49 (2), 396–403. 10.1007/s12020-014-0501-x 25522723

[B120] Momenzadeh S. Zamani S. Pourteymourfard-Tabrizi Z. Barreiro C. Jami M. S. (2021). Muscles proteome analysis; irisin administration mimics some molecular effects of exercise in quadriceps muscle. Biochimie 189, 144–157. 10.1016/j.biochi.2021.06.016 34217820

[B121] Moon H. Y. Becke A. Berron D. Becker B. Sah N. Benoni G. (2016). Running-induced systemic cathepsin B secretion is associated with memory function. Cell metab. 24 (2), 332–340. 10.1016/j.cmet.2016.05.025 27345423 PMC6029441

[B122] Munive V. Santi A. Torres-Aleman I. (2016). A concerted action of estradiol and insulin like growth factor I underlies sex differences in mood regulation by exercise. Sci. Rep. 6, 25969. 10.1038/srep25969 27170462 PMC4864325

[B123] Munive V. Zegarra-Valdivia J. A. Herrero-Labrador R. Fernandez A. M. Aleman I. T. (2019). Loss of the interaction between estradiol and insulin-like growth factor I in brain endothelial cells associates to changes in mood homeostasis during peri-menopause in mice. Aging 11 (1), 174–184. 10.18632/aging.101739 30636168 PMC6339786

[B124] Muñoz-Cánoves P. Scheele C. Pedersen B. K. Serrano A. L. (2013). Interleukin-6 myokine signaling in skeletal muscle: a double-edged sword? FEBS J. 280 (17), 4131–4148. 10.1111/febs.12338 23663276 PMC4163639

[B125] Nakajima S. Ohsawa I. Ohta S. Ohno M. Mikami T. (2010). Regular voluntary exercise cures stress-induced impairment of cognitive function and cell proliferation accompanied by increases in cerebral IGF-1 and GST activity in mice. Behav. brain Res. 211 (2), 178–184. 10.1016/j.bbr.2010.03.028 20307585

[B126] Ni J. Lan F. Xu Y. Nakanishi H. Li X. (2022). Extralysosomal cathepsin B in central nervous system: mechanisms and therapeutic implications. Brain pathol. Zurich, Switz. 32 (5), e13071. 10.1111/bpa.13071 PMC9425006 35411983

[B127] Nishijima T. Okamoto M. Matsui T. Kita I. Soya H. (2012). Hippocampal functional hyperemia mediated by NMDA receptor/NO signaling in rats during mild exercise. J. Appl. physiology (Bethesda, Md, 1985) 112 (1), 197–203. 10.1152/japplphysiol.00763.2011 21940846

[B128] Norling A. M. Gerstenecker A. T. Buford T. W. Khan B. Oparil S. Lazar R. M. (2020). The role of exercise in the reversal of IGF-1 deficiencies in microvascular rarefaction and hypertension. GeroScience 42 (1), 141–158. 10.1007/s11357-019-00139-2 31808026 PMC7031491

[B129] Oh K. J. Lee D. S. Kim W. K. Han B. S. Lee S. C. Bae K. H. (2016). Metabolic adaptation in obesity and type II diabetes: myokines, adipokines and hepatokines. Int. J. Mol. Sci. 18 (1), 8. 10.3390/ijms18010008 28025491 PMC5297643

[B130] Omura T. Sano M. Omura K. Hasegawa T. Doi M. Sawada T. (2005). Different expressions of BDNF, NT3, and NT4 in muscle and nerve after various types of peripheral nerve injuries. J. Peripher. Nerv. Syst. JPNS 10 (3), 293–300. 10.1111/j.1085-9489.2005.10307.x 16221288

[B131] Pan W. Banks W. A. Fasold M. B. Bluth J. Kastin A. J. (1998). Transport of brain-derived neurotrophic factor across the blood-brain barrier. Neuropharmacology 37 (12), 1553–1561. 10.1016/s0028-3908(98)00141-5 9886678

[B132] Pang B. P. S. Chan W. S. Chan C. B. (2021). Mitochondria homeostasis and oxidant/antioxidant balance in skeletal muscle-do myokines play a role? Antioxidants Basel, Switz. 10 (2), 179. 10.3390/antiox10020179 PMC7911667 33513795

[B133] Peake J. M. Della Gatta P. Suzuki K. Nieman D. C. (2015). Cytokine expression and secretion by skeletal muscle cells: regulatory mechanisms and exercise effects. Exerc. Immunol. Rev. 21, 8–25. 25826432

[B134] Pearson-Leary J. McNay E. C. (2016). Novel roles for the insulin-regulated glucose transporter-4 in hippocampally dependent memory. J. Neurosci. official J. Soc. Neurosci. 36 (47), 11851–11864. 10.1523/JNEUROSCI.1700-16.2016 PMC5125244 27881773

[B135] Pedersen B. K. (2009). Edward F. Adolph distinguished lecture: muscle as an endocrine organ: IL-6 and other myokines. J. Appl. physiology (Bethesda, Md, 1985) 107 (4), 1006–1014. 10.1152/japplphysiol.00734.2009 19696361

[B136] Pedersen B. K. (2019). Physical activity and muscle-brain crosstalk. Nat. Rev. Endocrinol. 15 (7), 383–392. 10.1038/s41574-019-0174-x 30837717

[B137] Pedersen B. K. Akerström T. C. Nielsen A. R. Fischer C. P. (2007). Role of myokines in exercise and metabolism. J. Appl. physiology (Bethesda, Md, 1985) 103 (3), 1093–1098. 10.1152/japplphysiol.00080.2007 17347387

[B138] Pedersen B. K. Febbraio M. A. (2008). Muscle as an endocrine organ: focus on muscle-derived interleukin-6. Physiol. Rev. 88 (4), 1379–1406. 10.1152/physrev.90100.2007 18923185

[B139] Pervaiz N. Hoffman-Goetz L. (2012). Immune cell inflammatory cytokine responses differ between central and systemic compartments in response to acute exercise in mice. Exerc. Immunol. Rev. 18, 142–157. 22876726

[B140] Petersen A. M. Pedersen B. K. (2006). The role of IL-6 in mediating the anti-inflammatory effects of exercise. J. physiology Pharmacol. official J. Pol. Physiological Soc. 57 (Suppl. 10), 43–51. 17242490

[B141] Phillips C. Fahimi A. (2018). Immune and neuroprotective effects of physical activity on the brain in depression. Front. Neurosci. 12, 498. 10.3389/fnins.2018.00498 30093853 PMC6070639

[B142] Prochnik A. Burgueño A. L. Rubinstein M. R. Marcone M. P. Bianchi M. S. Gonzalez Murano M. R. (2022). Sexual dimorphism modulates metabolic and cognitive alterations under HFD nutrition and chronic stress exposure in mice. Correlation between spatial memory impairment and BDNF mRNA expression in hippocampus and spleen. Neurochem. Int. 160, 105416. 10.1016/j.neuint.2022.105416 36055604

[B143] Puigserver P. Wu Z. Park C. W. Graves R. Wright M. Spiegelman B. M. (1998). A cold-inducible coactivator of nuclear receptors linked to adaptive thermogenesis. Cell 92 (6), 829–839. 10.1016/s0092-8674(00)81410-5 9529258

[B144] Radka S. F. Holst P. A. Fritsche M. Altar C. A. (1996). Presence of brain-derived neurotrophic factor in brain and human and rat but not mouse serum detected by a sensitive and specific immunoassay. Brain Res. 709 (1), 122–301. 10.1016/0006-8993(95)01321-0 8869564

[B145] Rai M. Demontis F. (2022). Muscle-to-Brain signaling via myokines and myometabolites. Brain plast. Amst. Neth. 8 (1), 43–63. 10.3233/BPL-210133 PMC9661353 36448045

[B146] Ransome M. I. Hannan A. J. (2013). Impaired basal and running-induced hippocampal neurogenesis coincides with reduced Akt signaling in adult R6/1 HD mice. Mol. Cell. Neurosci. 54, 93–107. 10.1016/j.mcn.2013.01.005 23384443

[B147] Reisman E. G. Hawley J. A. Hoffman N. J. (2024). Exercise-regulated mitochondrial and nuclear signalling networks in skeletal muscle. Sports Med. Auckl. N.Z. 54 (5), 1097–1119. 10.1007/s40279-024-02007-2 PMC11127882 38528308

[B148] Reza M. M. Subramaniyam N. Sim C. M. Ge X. Sathiakumar D. McFarlane C. (2017). Irisin is a pro-myogenic factor that induces skeletal muscle hypertrophy and rescues denervation-induced atrophy. Nat. Commun. 8 (1), 1104. 10.1038/s41467-017-01131-0 29062100 PMC5653663

[B149] Rezaee Z. Marandi S. M. Alaei H. Esfarjani F. (2023). Exercise-induced Brain-derived neurotrophic factor regulation in the brain dysfunctions. Sci. and Sports 38 (5-6), 519–526. 10.1016/j.scispo.2022.08.003

[B150] Roca-Rivada A. Al-Massadi O. Castelao C. Senín L. L. Alonso J. Seoane L. M. (2012). Muscle tissue as an endocrine organ: comparative secretome profiling of slow-oxidative and fast-glycolytic rat muscle explants and its variation with exercise. J. proteomics 75 (17), 5414–5425. 10.1016/j.jprot.2012.06.037 22800642

[B151] Rose-John S. (2012). IL-6 trans-signaling via the soluble IL-6 receptor: importance for the pro-inflammatory activities of IL-6. Int. J. Biol. Sci. 8 (9), 1237–1247. 10.7150/ijbs.4989 23136552 PMC3491447

[B152] Rose-John S. Winthrop K. Calabrese L. (2017). The role of IL-6 in host defence against infections: immunobiology and clinical implications. Nat. Rev. Rheumatol. 13 (7), 399–409. 10.1038/nrrheum.2017.83 28615731

[B153] Schmitz J. Gilberg E. Löser R. Bajorath J. Bartz U. Gütschow M. (2019). Cathepsin B: active site mapping with peptidic substrates and inhibitors. Bioorg. and Med. Chem. 27 (1), 1–15. 10.1016/j.bmc.2018.10.017 30473362

[B154] Schöbitz B. de Kloet E. R. Sutanto W. Holsboer F. (1993). Cellular localization of interleukin 6 mRNA and interleukin 6 receptor mRNA in rat brain. Eur. J. Neurosci. 5 (11), 1426–1435. 10.1111/j.1460-9568.1993.tb00210.x 8287192

[B155] Shahabi S. Esfarjani F. Reisi J. Momenzadeh S. Jami M. S. Zamani S. (2021). The effects of 8-week resistance and endurance trainings on bone strength compared to irisin injection protocol in mice. Adv. Biomed. Res. 10, 40. 10.4103/abr.abr_220_20 35071108 PMC8744425

[B156] Sharif K. Watad A. Bragazzi N. L. Lichtbroun M. Amital H. Shoenfeld Y. (2018). Physical activity and autoimmune diseases: get moving and manage the disease. Autoimmun. Rev. 17 (1), 53–72. 10.1016/j.autrev.2017.11.010 29108826

[B157] So B. Kim H. J. Kim J. Song W. (2014). Exercise-induced myokines in health and metabolic diseases. Integr. Med. Res. 3 (4), 172–179. 10.1016/j.imr.2014.09.007 28664094 PMC5481763

[B158] Sølvsten C. A. E. de Paoli F. Christensen J. H. Nielsen A. L. (2018). Voluntary physical exercise induces expression and epigenetic remodeling of VegfA in the rat Hippocampus. Mol. Neurobiol. 55 (1), 567–582. 10.1007/s12035-016-0344-y 27975171

[B159] Sorrells S. F. Paredes M. F. Cebrian-Silla A. Sandoval K. Qi D. Kelley K. W. (2018). Human hippocampal neurogenesis drops sharply in children to undetectable levels in adults. Nature 555 (7696), 377–381. 10.1038/nature25975 29513649 PMC6179355

[B160] Souza P. S. Gonçalves E. D. Pedroso G. S. Farias H. R. Junqueira S. C. Marcon R. (2017). Physical exercise attenuates experimental autoimmune encephalomyelitis by inhibiting peripheral immune response and blood-brain barrier disruption. Mol. Neurobiol. 54 (6), 4723–4737. 10.1007/s12035-016-0014-0 27447807

[B161] Tanimura R. Kobayashi L. Shirai T. Takemasa T. (2022). Effects of exercise intensity on white adipose tissue browning and its regulatory signals in mice. Physiol. Rep. 10 (5), e15205. 10.14814/phy2.15205 35286020 PMC8919700

[B162] Tao Y. S. Piao S. G. Jin Y. S. Jin J. Z. Zheng H. L. Zhao H. Y. (2018). Expression of brain-derived neurotrophic factor in kidneys from normal and cyclosporine-treated rats. BMC Nephrol. 19 (1), 63. 10.1186/s12882-018-0852-2 29540150 PMC5853162

[B163] Trillaud E. Klemmer P. Malin S. K. Erdbrügger U. (2023). Tracking biomarker responses to exercise in hypertension. Curr. Hypertens. Rep. 25 (10), 299–311. 10.1007/s11906-023-01252-6 37428393 PMC10505098

[B164] Turk V. Stoka V. Vasiljeva O. Renko M. Sun T. Turk B. (2012). Cysteine cathepsins: from structure, function and regulation to new frontiers. Biochimica biophysica acta 1824 (1), 68–88. 10.1016/j.bbapap.2011.10.002 PMC7105208 22024571

[B165] Vajdos F. F. Ultsch M. Schaffer M. L. Deshayes K. D. Liu J. Skelton N. J. (2001). Crystal structure of human insulin-like growth factor-1: detergent binding inhibits binding protein interactions. Biochemistry 40 (37), 11022–11029. 10.1021/bi0109111 11551198

[B167] Vallières L. Campbell I. L. Gage F. H. Sawchenko P. E. (2002). Reduced hippocampal neurogenesis in adult transgenic mice with chronic astrocytic production of interleukin-6. J. Neurosci. official J. Soc. Neurosci. 22 (2), 486–492. 10.1523/JNEUROSCI.22-02-00486.2002 PMC6758670 11784794

[B168] Vallières L. Lacroix S. Rivest S. (1997). Influence of interleukin-6 on neural activity and transcription of the gene encoding corticotrophin-releasing factor in the rat brain: an effect depending upon the route of administration. Eur. J. Neurosci. 9 (7), 1461–1472. 10.1111/j.1460-9568.1997.tb01500.x 9240403

[B169] Vannucci S. J. Koehler-Stec E. M. Li K. Reynolds T. H. Clark R. Simpson I. A. (1998). GLUT4 glucose transporter expression in rodent brain: effect of diabetes. Brain Res. 797 (1), 1–11. 10.1016/s0006-8993(98)00103-6 9630471

[B170] Villena J. A. (2015). New insights into PGC-1 coactivators: redefining their role in the regulation of mitochondrial function and beyond. FEBS J. 282 (4), 647–672. 10.1111/febs.13175 25495651

[B171] Vints W. A. J. Levin O. Fujiyama H. Verbunt J. Masiulis N. (2022). Exerkines and long-term synaptic potentiation: mechanisms of exercise-induced neuroplasticity. Front. Neuroendocrinol. 66, 100993. 10.1016/j.yfrne.2022.100993 35283168

[B172] Vivar C. Potter M. C. van Praag H. (2013). All about running: synaptic plasticity, growth factors and adult hippocampal neurogenesis. Curr. Top. Behav. Neurosci. 15, 189–210. 10.1007/7854_2012_220 22847651 PMC4565722

[B173] Wang B. Liang J. Lu C. Lu A. Wang C. (2024). Exercise regulates myokines in aging-related diseases through muscle-brain crosstalk. Gerontology 70 (2), 193–209. 10.1159/000535339 38008091

[B174] Wang B. L. Jin H. Han X. Q. Xia Y. Liu N. F. (2018). Involvement of brain-derived neurotrophic factor in exercise-induced cardioprotection of post-myocardial infarction rats. Int. J. Mol. Med. 42 (5), 2867–2880. 10.3892/ijmm.2018.3841 30226568

[B175] Wang J. Zheng M. Yang X. Zhou X. Zhang S. (2023). The role of cathepsin B in pathophysiologies of non-tumor and tumor tissues: a systematic review. J. Cancer 14 (12), 2344–2358. 10.7150/jca.86531 37576397 PMC10414043

[B176] Weinstein G. Beiser A. S. Choi S. H. Preis S. R. Chen T. C. Vorgas D. (2014). Serum brain-derived neurotrophic factor and the risk for dementia: the Framingham Heart Study. JAMA neurol. 71 (1), 55–61. 10.1001/jamaneurol.2013.4781 24276217 PMC4056186

[B177] Wende A. R. Schaeffer P. J. Parker G. J. Zechner C. Han D. H. Chen M. M. (2007). A role for the transcriptional coactivator PGC-1alpha in muscle refueling. J. Biol. Chem. 282 (50), 36642–36651. 10.1074/jbc.M707006200 17932032

[B178] Wrann C. D. White J. P. Salogiannnis J. Laznik-Bogoslavski D. Wu J. Ma D. (2013). Exercise induces hippocampal BDNF through a PGC-1α/FNDC5 pathway. Cell metab. 18 (5), 649–659. 10.1016/j.cmet.2013.09.008 24120943 PMC3980968

[B179] Yang X. Brobst D. Chan W. S. Tse M. C. L. Herlea-Pana O. Ahuja P. (2019). Muscle-generated BDNF is a sexually dimorphic myokine that controls metabolic flexibility. Sci. Signal. 12 (594), eaau1468. 10.1126/scisignal.aau1468 31409756 PMC7219567

[B180] Yardimci A. Ertugrul N. U. Ozgen A. Ozbeg G. Ozdede M. R. Ercan E. C. (2023). Effects of chronic irisin treatment on brain monoamine levels in the hypothalamic and subcortical nuclei of adult male and female rats: an HPLC-ECD study. Neurosci. Lett. 806, 137245. 10.1016/j.neulet.2023.137245 37061025

[B181] Yau S. Y. Lau B. W. Zhang E. D. Lee J. C. Li A. Lee T. M. (2012). Effects of voluntary running on plasma levels of neurotrophins, hippocampal cell proliferation and learning and memory in stressed rats. Neuroscience 222, 289–301. 10.1016/j.neuroscience.2012.07.019 22813995

[B182] Yoon M. C. Hook V. O'Donoghue A. J. (2022). Cathepsin B dipeptidyl carboxypeptidase and endopeptidase activities demonstrated across a broad pH range. Biochemistry 61 (17), 1904–1914. 10.1021/acs.biochem.2c00358 35981509 PMC9454093

[B183] Yoshida T. Delafontaine P. (2020). Mechanisms of IGF-1-mediated regulation of skeletal muscle hypertrophy and atrophy. Cells 9 (9), 1970. 10.3390/cells9091970 32858949 PMC7564605

[B184] Yuan D. Zhao Y. Banks W. A. Bullock K. M. Haney M. Batrakova E. (2017). Macrophage exosomes as natural nanocarriers for protein delivery to inflamed brain. Biomaterials 142, 1–12. 10.1016/j.biomaterials.2017.07.011 28715655 PMC5603188

[B185] Zakharova A. N. Kironenko T. A. Milovanova K. G. Orlova A. A. Dyakova E. Y. Kalinnikova Yu G. (2021). Treadmill training effect on the myokines content in skeletal muscles of mice with a metabolic disorder model. Front. physiology 12, 709039. 10.3389/fphys.2021.709039 PMC8631430 34858197

[B186] Zhang L. Hu X. Luo J. Li L. Chen X. Huang R. (2013). Physical exercise improves functional recovery through mitigation of autophagy, attenuation of apoptosis and enhancement of neurogenesis after MCAO in rats. BMC Neurosci. 14, 46. 10.1186/1471-2202-14-46 23565939 PMC3637142

[B187] Zhang W. Guo Y. Li B. Zhang Q. Liu J. H. Gu G. J. (2018). GDF11 rejuvenates cerebrovascular structure and function in an animal model of alzheimer's disease. J. Alzheimer's Dis. JAD 62 (2), 807–819. 10.3233/JAD-170474 29480172

[B188] Zhang X. Xu S. Hu Y. Liu Q. Liu C. Chai H. (2023). Irisin exhibits neuroprotection by preventing mitochondrial damage in Parkinson's disease. NPJ Parkinson's Dis. 9 (1), 13. 10.1038/s41531-023-00453-9 36720890 PMC9889817

[B189] Zhang Y. Kang J. D. Zhao D. Ghosh S. S. Wang Y. Tai Y. (2021). Hepatic branch vagotomy modulates the gut-liver-brain Axis in murine cirrhosis. Front. physiology 12, 702646. 10.3389/fphys.2021.702646 PMC8268007 34248683

[B190] Zhao J. L. Jiang W. T. Wang X. Cai Z. D. Liu Z. H. Liu G. R. (2020). Exercise, brain plasticity, and depression. CNS Neurosci. and Ther. 26 (9), 885–895. 10.1111/cns.13385 32491278 PMC7415205

[B191] Zhou S. B. Xue M. Liu W. Chen Y. X. Chen Q. Y. Lu J. S. (2023). Age-related attenuation of cortical synaptic tagging in the ACC is rescued by BDNF or a TrkB receptor agonist in both sex of mice. Mol. brain 16 (1), 4. 10.1186/s13041-022-00992-x 36604761 PMC9817281

[B192] Zhou W. Barkow J. C. Freed C. R. (2017). Running wheel exercise reduces α-synuclein aggregation and improves motor and cognitive function in a transgenic mouse model of Parkinson's disease. PloS one 12 (12), e0190160. 10.1371/journal.pone.0190160 29272304 PMC5741244

[B193] Zuo Q. Qu F. Li N. Wang S. Liu J. Xu C. (2019). Eccentric exercise results in a prolonged increase in interleukin-6 and tumor necrosis factor-α levels in rat skeletal muscle. J. muscle Res. cell Motil. 40 (3-4), 379–387. 10.1007/s10974-019-09554-6 31520264

